# The polymyxin B-induced transcriptomic response of a clinical, multidrug-resistant *Klebsiella pneumoniae* involves multiple regulatory elements and intracellular targets

**DOI:** 10.1186/s12864-016-3070-y

**Published:** 2016-10-25

**Authors:** Pablo Ivan Pereira Ramos, Márlon Grégori Flores Custódio, Guadalupe del Rosario Quispe Saji, Thiago Cardoso, Gisele Lucchetti da Silva, Graziela Braun, Willames M. B. S. Martins, Raquel Girardello, Ana Tereza Ribeiro de Vasconcelos, Elmer Fernández, Ana Cristina Gales, Marisa Fabiana Nicolás

**Affiliations:** 1 Laboratório Nacional de Computação Científica, Petrópolis, Rio de Janeiro Brazil; 2 Centro de Pesquisas Gonçalo Moniz, FIOCRUZ, Salvador, Bahia Brazil; 3 Laboratório Alerta. Division of Infectious Diseases, Department of Internal Medicine, Escola Paulista de Medicina/Universidade Federal de São Paulo, São Paulo, São Paulo Brazil; 4 Facultad de Ingeniería, Universidad Católica de Córdoba, CONICET, Córdoba, Argentina

**Keywords:** Antibiotic resistance, *Klebsiella pneumoniae*, Pathogen, Polymyxin B, RNA-seq, Transcriptomics

## Abstract

**Background:**

The emergence of multidrug-resistant *Klebsiella pneumoniae* is a major public health concern. Many *K. pneumoniae* infections can only be treated when resorting to last-line drugs such as polymyxin B (PB). However, resistance to this antibiotic is also observed, although insufficient information is described on its mode of action as well as the mechanisms used by resistant bacteria to evade its effects. We aimed to study PB resistance and the influence of abiotic stresses in a clinical *K. pneumoniae* strain using whole transcriptome profiling.

**Results:**

We sequenced 12 cDNA libraries of *K. pneumoniae* Kp13 bacteria, from two biological replicates of the original strain Kp13 (Kp13) and five derivative strains: induced high-level PB resistance in acidic pH (Kp13_pH_), magnesium deprivation (Kp13_Mg_), high concentrations of calcium (Kp13_Ca_) and iron (Kp13_Fe_), and a control condition with PB (Kp13_PolB_). Our results show the involvement of multiple regulatory loci that differentially respond to each condition as well as a shared gene expression response elicited by PB treatment, and indicate the participation of two-regulatory components such as ArcA-ArcB, which could be involved in re-routing the *K. pneumoniae* metabolism following PB treatment. Modules of co-expressed genes could be determined, which correlated to growth in acid stress and PB exposure. We hypothesize that polymyxin B induces metabolic shifts in *K. pneumoniae* that could relate to surviving against the action of this antibiotic.

**Conclusions:**

We obtained whole transcriptome data for *K. pneumoniae* under different environmental conditions and PB treatment. Our results supports the notion that the *K. pneumoniae* response to PB exposure goes beyond damaged membrane reconstruction and involves recruitment of multiple gene modules and intracellular targets.

**Electronic supplementary material:**

The online version of this article (doi:10.1186/s12864-016-3070-y) contains supplementary material, which is available to authorized users.

## Background

Antibiotic discovery represented one of the most significant events in human health improvement. The antibiotic ‘golden age’, when many classes were identified and commercialized, extended from 1950 to 1960 [1]. Parallel to antibiotic availability, there were ever increasing numbers of bacteria that could overcome treatment and displayed a resistant phenotype. Extreme cases involve bacteria that display resistance against all clinically available agents [2]. Although the emergence of resistance pre-dates the antibiotic era, widespread multi-drug resistance (MDR) is now a major concern that limit therapeutic options in the treatment of many bacterial infections.

Polymyxins, including polymyxin B and colistin, are cationic antimicrobial lipopeptides (CAMPs) that have been available for decades, but due to their nephrotoxicity and the lack of pharmacodynamics and pharmacokinetics information, have had its use greatly diminished [3, 4]. The mode of action of these drugs is also not completely elucidated, but generally involves interaction with lipopolysaccharides (LPS) located in the outer membrane of Gram-negative bacteria, competing with the calcium and magnetic ions that stabilize LPS, allowing for drug uptake to the cell interior [3]. It has been reported that PB activity may involve inhibition of vital respiratory enzymes located in the inner membrane, such as NADH-quinone oxidoreductase (NDH-2) [5], thereby hampering bacterial respiration.

Recently, polymyxins have re-emerged, now as ‘last resort’ drugs, used in patients with difficult-to-treat infections caused by MDR Gram-negative bacteria including *K. pneumoniae*, *Pseudomonas aeruginosa* and *Acinetobacter baumannii.* Resistance to polymyxins, however, has also been described in many settings [6, 7].

In order to better understand the mechanisms employed by MDR bacteria to evade the action of polymyxins, we applied whole transcriptomic profiling of a clinical *K. pneumoniae* strain with induced, high-level resistance to polymyxin B. We previously reported the complete genome of this strain [8], and we identified that it has a truncated *mgrB* gene, consistent with reports that mutations in this gene represent one of the various mechanisms for acquired colistin resistance [9–11]. Despite Kp13 being already resistant to polymyxin B, we aimed to induce additional adaptive responses by growth in high polymyxin B concentrations while also probing in vitro the effects of diverse environmental stimuli. Variations in abiotic stimuli govern many transcriptional regulatory responses, including in two-component regulatory systems (TCRSs) that may play a role in drug resistance. For instance, varying concentrations of divalent cations (such as Mg^2+^ and Ca^2+^) alter the expression of important global regulators such as the PhoP-PhoQ TCRS, which regulates over 40 genes and has been implicated in colistin resistance [12]. On the other hand, acid pH and iron supplementation were shown to induce colistin resistance in *A. baumannii* probably involving alterations in regulatory circuits [13].

While genome analyses provide a complete picture of the genes present (and potentially expressed), only through the use of high throughput expression profiling techniques such as RNA sequencing (RNA-seq), as performed in this report, can we find consistent expression patterns that can aid in pinpointing the intracellular targets and metabolic processes related to polymyxin B mode of action and resistance.

## Methods

### Bacterial strain, higher-level polymyxin B resistance induction and growth conditions

*Klebsiella pneumoniae* subsp. *pneumoniae* Kp13 (hereafter referred as Kp13) was isolated in 2009 from a patient during an event of nosocomial outbreak due to KPC-2-producing *K. pneumoniae* bacteria in an intensive care unit from the Hospital Universitário (UEL), South Brazil. The complete genome of this strain is comprised by one chromosome and six plasmids and was completely sequenced by our group in a previous work [8]. Kp13 harbors multiple virulence and resistance determinants in its genome, and presents resistant to many antibiotics [8, 14].

Kp13 was originally resistant to polymyxin B (PB) [8] at a minimum inhibitory concentration (MIC) of 32 μg mL^−1^ (EUCAST breakpoint [15]). We have induced an increased, high-level resistance to this antibiotic by growing the bacteria in solid Luria-Bertani medium (LB, Oxoid, Basingstoke, England) in the presence of crescent polymyxin B (Sigma-Aldrich, St. Louis, MO, USA) concentrations and passaging the bacteria in serial dilutions of PB beginning with a concentration of 8 μg mL^−1^ up to 64 μg mL^−1^. Before and after the induction of resistance, PB MICs were confirmed by CLSI broth microdilutions.

Then, we selected from the original strain *K. pneumoniae* Kp13 five derivative strains subcultured in different physical conditions, as shown in Table 1. We note that the original and derivate strains represented a single clone presenting high-level resistance to polymyxin B with MIC >32 μg mL^−1^ and MIC >64 μg mL^−1^, respectively (Table 1). Pulsed-field gel electrophoresis performed in these six strains (original and derivatives, data not shown), resulted in similar genetic profiles suggestive that expression mechanisms may play an important role to their distinct resistance phenotypes.

**Table 1:** Strains used in RNA sequencing experiments and respective growth conditions

Condition name	Strain	Conditions^a^	Concentration (mg L^−1^)			pH	MIC^b^ (μg mL^−1^)	Reference
			CaCl_2_	MgCl_2_	FeCl_2_			
Non-induced	Kp13	Original strain, no polymyxin B supplementation	25.0	12.5	0.0	7.0	32	[8]
PB	Kp13_PolB_	LB supplemented with 4 μg mL^−1^ of polymyxin B	25.0	12.5	0.0	7.0	64	This study
PB + high calcium	Kp13_Ca_	LB supplemented with 4 μg mL^−1^ of polymyxin B plus high [Ca^2+^]	75.0	12.5	0.0	7.0	64	This study
PB + no magnesium	Kp13_Mg_	LB supplemented with 4 μg mL^−1^ of polymyxin B without Mg^2+^	25.0	0.0	0.0	7.0	128	This study
PB + high iron	Kp13_Fe_	LB supplemented with 4 μg mL^−1^ of polymyxin B plus high [Fe^2+^]	25.0	12.5	75.0	7.0	64	This study
PB + low pH	Kp13_pH_	LB supplemented with 4 μg mL^−1^ of polymyxin B in acid pH	25.0	12.5	0.0	5.8	64	This study

^a^The concentration of polymyxin corresponded to the concentration used during the extraction of RNA for RNAseq. All experiments were performed in duplicate. ^b^,Minimum inhibitory concentration of polymyxin B in the respective strain

### Total RNA extraction and Illumina sequencing

For each condition, bacterial cultures were grown until final-log phase and cells were all harvested at OD 600 nm. Total RNA extraction was performed using RNeasy Mini Kit (Qiagen) with DNAse treatment (Qiagen). Enrichment for mRNA was performed using the MICROBExpress Bacterial mRNA Purification (Ambion), and rRNA were removed using Ribo-Zero kit (Epicentre). Sequencing of two biological replicates per condition was performed in an Illumina HiSeq 2500 (Fasteris, Geneva, Switzerland) using a paired-end strategy.

### Expression validation by real time PCR

To validate the expression of selected genes, the transcriptional levels of TCRS components related with polymyxin B resistance were evaluated by quantitative real-time PCR (qRT-PCR). Total RNA was isolated from *K. pneumoniae* Kp13 and derivative strains subcultured in distinct conditions (Table 1) using the RNeasy Mini Kit (Qiagen, Hilden, Germany) with addition of RNase-free DNase (Qiagen, Hilden, Germany). Reverse transcription of the extracted RNA was performed using the High Capacity cDNA Reverse Transcription Kit (Life Technologies, Carlsbad, CA, USA). The pairs of primers used for the amplification of the *arcB, pmrB, phoP* and 16S rDNA genes are shown in Table 2. Transcripts were quantified in triplicate using SYBR® Green PCR Master Mix (Life Technologies, Carlsbad, CA, USA) in the 7500 Real Time system (Life Technologies, Carlsbad, CA, USA). The magnitude of expression (ΔCT) of the studied gene was then obtained by normalization against the housekeeping 16S gene. The equation 2^-ΔΔCT^, where ΔΔCT = ΔCT_target_ − ΔCT_reference_, was applied for relative quantification (RQ) of the differences in gene expression levels between two conditions. To calculate the RQ of each studied gene the expression at each probed condition (PB+high calcium; PB+high iron; PB+no magnesium; and PB+low pH, with the respective strains as in Table 1) was normalized against the PB condition (Table 1).

**Table 2:** Primers sequence used in qRT-PCR experiments

Primers	Sequence	Amplicon size	Reference
arcB_RT-F	5′ GCTGAACGTCCAACTGAAAG 3′	157 bp	This study
arcB_RT-R	5′ GGAGGATTGCTGTTCGAGC 3′		
phoP_RT-F	5′ TGCCGGATGAAGACGGACTA 3′	226 bp	This study
phoP_RT-R	5′ AGGGAGATCACCTGTGAGGC 3′		
pmrB_RT-F	5′ GCTGATCCAGCGTCTCGATC 3′	106 bp	This study
pmrB_RT-R	5′ CAACAGCACCTGCTGGTAGC 3′		
16S_RT-F	5′ CAGCTCGTGTCGTGAGATGT 3′	150 bp	[66]
16S_RT-R	5′ CGTAAGGGCCATGATGACTT 3′		

### Growth curve and analysis of pH variance

Strains Kp13_PolB_ and Kp13 (non-induced) were subcultivated in the absence or presence of polymyxin B to evaluate differences in bacterial fitness. The growth of bacterial strains was monitored until stationary phase of growth was reached. The strains were cultured in the absence or presence of polymyxin B sulfate 4 μg mL^−1^ in LB broth overnight. Three milliliters of each culture were diluted in 447 mL of fresh LB broth in absence or presence of polymyxin B, and incubated under constant shaking at 37 °C. Ten milliliters of each bacterial suspension was removed every hour. One milliliter was transferred to a cuvette for turbidity measure at 600 nm wavelength, while the remaining 9 mL were used to quantify the pH variation during the bacteria growth.

### Data analysis

Raw read preprocessing and quality control (QC) were performed with FastQC [16], which allows assessment of diverse quality metrics of the sequencing data. Removal of adapters and low-quality reads were performed with Skewer [17]. We applied the normalization method Removal of Unwanted Variation (RUV) in order to properly correct for batch effects in experimental data obtained through RNA sequencing [18]. For this approach we used the MLST genes of *Klebsiella*, available at the Institut Pasteur MLST database website [19], as control genes among the cDNA libraries data [20]. Reads passing QC were aligned to the *K. pneumoniae* Kp13 reference genome (NCBI BioProject accession PRJNA78291) using Top-Hat2 [21], and SAMtools [22] was used to parse alignments and QC of the mappings. Gene counts were obtained using HTSeq [23] and differential gene expression was evaluated with edgeR [24], which models expression data as negative binomial distributed. The PB condition (Table 1) was the reference condition for comparisons performed in the RNA-seq analyses unless otherwise noted in the text.

### Co-expression network construction

Gene co-expression networks were created using weighted gene co-expression network analysis (WGCNA) package in R [25]. Variance-stabilized count data were used as a measure of gene expression. The biweight midcorrelation algorithm implemented in the *bicor* function in WGCNA was used to calculate expression correlation between each pair of genes. This correlation measure is similar to Pearson’s correlation with the advantage of being more robust to outliers [26]. The correlation matrix was transformed into a weighted adjacency matrix representing connection strengths using a soft-thresholding approach by applying a power transformation with an appropriate β parameter, f(*x*) = *x* ^β^. β is chosen such that the resulting adjacency matrix leads to a weighted co-expression network that displays approximately scale-free topology. In this framework, a value of β = 14 was used in our analyses, which resulted in a scale-free model fitting with *R* ^2^ >0.80. Once the adjacency matrix was constructed, we derived from it a topological overlap matrix (TOM), which takes gene expression connectivity into account. 1-TOM was used as dissimilarity measure for hierarchical clustering and module detection. Module assignment was determined using the dynamic tree cut algorithm within WGCNA [25]. Module eigengenes were tested for association with the different experimental conditions and those displaying significant correlation, defined as Pearson's |*r|* ≥ 0.6; *p*-value <0.05, were more thoroughly studied by means of Gene Ontology (GO) enrichment analysis.

### GO enrichment analysis

Genes belonging to each module of interest were submitted to ClueGO v. 2.2.4 [27] for Cytoscape v.3.3.0 [28], which performs Gene Ontology enrichment analysis and cluster detection. Only biological process terms were considered, and GO terms were grouped if their kappa score was ≥0.4. The statistical test chosen to identify enriched biological process terms was the right-sided hypergeometric test, considering as significant terms with adjusted *p*-value <0.05 and comprising at least three genes. The Benjamini-Hochberg procedure was applied to control the false discovery rate. To study both the more general biological processes associated to each module as well as the more specific terms, the “GO tree interval” parameter was set to either (*min* = 2, *max* = 5) or (*min* = 7, *max* = 15), respectively.

## Results and discussion

### Evaluation of the global transcriptional response of *K. pneumoniae* to increased polymyxin B resistance

Firstly, we investigated the general transcriptional response of *K. pneumoniae* Kp13 in view of increased resistance to polymyxin B (PB). Prior to high-level experimental induction of resistance, Kp13 was already PB-resistant (MIC 32 μg mL^−1^) and had a truncated *mgrB* gene due to transposase insertion, a mechanism already described in the literature as providing PB resistance [9, 11]. We were interested in detecting additional mechanisms of resistance to this antibiotic as well as understand the effect of PB treatment combined with diverse abiotic stresses in the global gene expression response of *K. pneumoniae.*

**Fig. 1:**
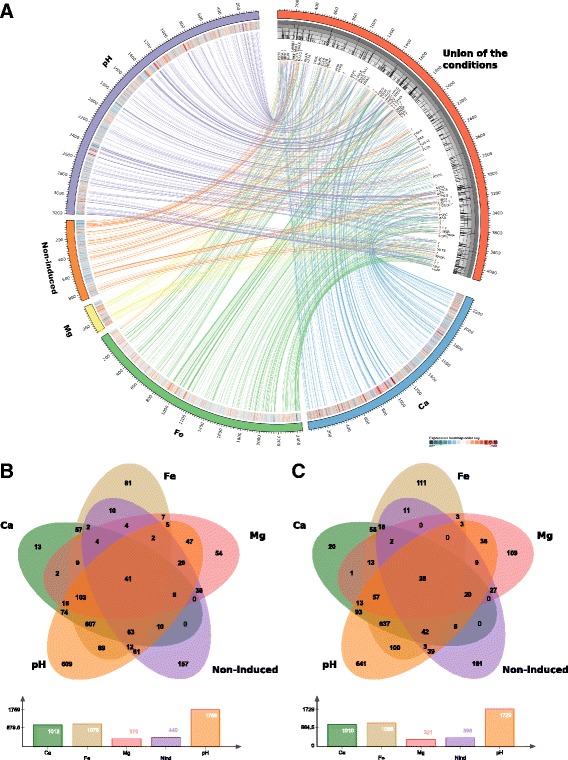
Transcriptomic response of *Klebsiella pneumoniae* Kp13 to different environmental stimuli. **a** The *outer rings* organized as pseudo-chromosomes harbor differentially expressed genes respective to the PB condition, except for the *red ring* representing the union of the conditions. Links from the top 10 % DE (up- and down-regulated) genes to the union of conditions are depicted, and for each condition a heatmap of expression of each gene (in log_2_[fold-change] scale) is shown. Below the union of the conditions an histogram of the frequency of DE genes is shown (i.e. if a gene appears DE in all conditions a value of five is shown in the histogram). Selected gene labels are shown on the *innermost ring*, *question marks* denote hypothetical genes being found differentially expressed. **b**, **c** Venn diagram showing the differentially expressed up- (Panel *b*) and down-regulated (Panel *c*) genes in the different conditions relative to PB. Criteria for significance: FDR≤0.01. The image was prepared with Circos [67] and jvenn [68]

A total of 12 cDNA libraries were generated that included the six experimental conditions (Table 1) each with two biological replicates. The total number of raw reads obtained was 73,438,485. The number of expressed genes ranged from 5613 to 5662, fairly stable among conditions. The presence of a large number of genes in the *K. pneumoniae* transcriptome is not unsual, as previous reports with multiple isolates found between 4744 and 5378 genes expressed, which is also dependent on the growth conditions [29]. The number of differentially expressed (DE) genes (relative to condition PB in Table 1; FDR≤0.01) were 838; 691; 2022; 2162 and 3488 DE genes in Kp13 strain (original strain), Kp13_Mg_ (PB+no magnesium), Kp13_Ca_ (PB+high calcium), Kp13_Fe_ (PB+high iron) and Kp13_pH_ (PB+low pH), respectively (Fig. 1a). We note the difference between the original strain Kp13 and the induction of a higher level resistance against polymyxin B in the other conditions (Table 1). The original strain as well as the Kp13_Mg_ strain were the least responsive, in terms of DE genes, relative to the PB condition. We also determined the genes with shared PB-dependent differential expression, which is composed of 41 up- and 28 down-regulated genes (Fig. 1b and c), by considering the gene expression in the Kp13_PolB_ strain relative to the strains grown in abiotic changes (Kp13 [to which no PB was added], Kp13_Ca_, Kp13_Fe_, Kp13_Mg_ and Kp13_pH_ [to which PB was added]). These can be regarded as the core, sustained activation/repression response to PB treatment independent of abiotic stresses (Additional file 1). On the other hand, the complete expression table of each gene relative to the PB condition is available as Additional file 2.

Among the shared PB-dependent up-regulated genes are various transcriptional factors (TFs). These TFs (and corresponding locus) include the OmpR regulator (KP13_00688) as well as the *argR* gene (KP13_31778) and genes encoding for a putative HTH-transcriptional regulator (KP13_03008; KP13_00251). Also detected was *arcB* (KP13_03225), part of the TCRS *arcAB* which has been shown to be highly pleiotropic, and modulates the expression of genes encoding for proteins with membrane modification functions as well as TCA cycle enzymes depending on oxygen levels or redox state [30, 31]. Other TFs detected included *rpoH* (KP13_08477), *rpoN* (KP13_03232). *rpoH,* encoding for the RNA polymerase σ^32^ factor is involved in stress response, mobilizing genes that constitute the heat-shock stimulon, such as those encoding for chaperones and proteases [32], while *rpoN* codes the σ^54^ factor, involved in the regulation of nitrogen utilization genes. Among the upregulated genes we also highlight *wzzE* (KP13_13130) and the ORF KP13_00225, encoding for a lipopolysaccharide biosynthesis protein and a putative inner membrane protein, respectively. Overall, the combined evidence show that the main up-regulated functions in PB treatment are those related to membrane functionality and maintenance. This is in line with the primary mode of action reported for this antibiotic, which permeabilizes the Gram-negative bacterial OM via direct interaction with the LPS [33].

Examination of down-regulated genes revealed that both the glycerol uptake (*glpF* and *glpT*) and metabolic pathways related to anaerobic catabolism of glycerol (*dhaD, dhaK, dhaM, glpA, glpB, glpC, glpD, glpQ* and *plsY*) are repressed by exposure to polymyxin B independently of environmental stimuli. Interestingly, a secondary mode of action for PB has been described and involves inhibitory activity against NDH-2, a vital respiratory enzyme in the bacterial inner membrane [5]. This would lead to the activation of genes involved in the response to decreased oxidative power within the cell, including *arcB*, as we have observed. Besides repressing TCA genes it is also known that the ArcA-ArcB TCRS acts repressing genes encoding primary dehydrogenases such as *glpD* [31], which would explain our findings.

### Respiratory/fermentative pathways and hydroxyl radical scavenging form part of the response of *K. pneumoniae* to polymyxin B and abiotic stimuli

*K. pneumoniae* is a metabolically versatile bacterium able to grow aerobically or anaerobically, and its genome contains all the genes encoding for the complete set of enzymes for obtaining energy from respiration or fermentation. How the bacteria adapt its metabolism into any of these growth conditions depends on the availability of oxygen (aerobic respiration) or alternative electron acceptors (e.g. nitrate, nitrite, fumarate during anaerobic respiration), and on the fermentability of the available carbon sources. The control between these different metabolic modes lies on the repression and derepression of the corresponding enzymes [34, 35]. Recent studies report that polymyxin B inhibits the activity of NDH-2 in the bacterial inner membrane of *K. pneumoniae*, *E. coli* and *A. baumannii* [5]. Besides the type II NDH-2 that is encoded by the *ndh* gene, these bacteria produce another type of NADH-quinone oxidoreductase for the respiratory chain, namely the type I (NDH-1) encoded by 13 tandemly arranged genes (*nuoA*-*nuoN*) [34]. In *E. coli* the NDH-2 is dominantly expressed under aerobic growth conditions, whereas the NDH-1 is induced under semiaerobic conditions [34]. So far, there are few reports on the effects of disruption of NADH-quinone oxidoreductases activity, particularly for NDH-2 in Gram-negative bacilli [5]. Presumably, in *A. baumannii* the mechanism of bacterial killing by polymyxins, which is mediated by release of hydroxyl radicals, might be due to disruption of a critical respiratory chain enzyme [36]. By our RNA-seq approach, *ndh* showed slightly decreased expression following antibiotic treatment (0.5-log-fold decrease with FDR≤0.01) as well as some genes encoding for NDH-1 (0.3-log-fold decrease significant differentially expressed genes at a FDR of <0.05) (Table 3). The only primary dehydrogenases (DH) showing increased gene expression after treatment with PB were formate hydrogenylases (*fdhF, hycA-hycH),* D-amino acid DH (*dad* or *dad2*), glucose DH (*gcD*) and some subunits of succinate DH (*sdhCDAB*) (Table 3). Regarding the expression of genes involved in the biosynthesis of ubiquinone (UQ) and naphthoquinone menaquinone (MK), our results show that, as much as NDH-1 and NDH-2, most were repressed after treatment with PB (Table 3). This result is in agreement with the knowledge that NADH dehydrogenases type I and II transfer electrons to UQ as well as to the naphthoquinones [37].

**Table 3:** Relative expression of quinone biosynthesis and oxidoreductase genes belonging to the *K. pneumoniae* Kp13 respiratory chain

Enzyme name	Gene name	Locus ID	log_2_FC
Primary dehydrogenases (DH):			
Formate dehydrogenase-N subunit alpha (FDH-N subunit alpha)	*fdnG*	KP13_32232	*0.1*
Formate dehydrogenase-N subunit beta (FDH-N subunit beta)	*fdnH*	KP13_04527	*−0.3*
Formate dehydrogenase-N subunit (FDH-N subunit gamma)	*fdnI*	KP13_04529	*0.1*
Formate hydrogenlyase H	*fdhF*	KP13_05320	**1.1**
Formate hydrogenlyase regulatory protein hycA	*hycA*	KP13_02572	**0.6**
Formate hydrogenlyase subunit 2 to 7	*hycB-G*	KP13_02573-8	**0.9, 1.6, 0.9, 0.8, 0.9, 1.2**
Formate hydrogenlyase maturation protein HycH	*hycH*	KP13_02579	**1.3**
NADH-quinone oxidoreductase (NDH-1)	*nuoA-nuoN*	KP13_00993-81	*0.1, 0, 0.2, 0, 0, −0.2, 0, −0.3, −0.1, −0.2, −0.1, −0.1, −0.1*
NADH-quinone oxidoreductase (NDH-2)	*ndh*	KP13_04904	**−0.5**
Glycerol-3-P DH_O_	*glpD*	KP13_00670	**−1.5**
Glycerol-3-P DH N (subunit A)	*glpA*	KP13_00962	**−3.6**
Glycerol-3-P DH N (subunit B)	*glpB*	KP13_00963	**−2.1**
Glycerol-3-P DH N (subunit C)	*glpC*	KP13_00964	**−1.4**
Pyruvate oxidase	*poxB*	KP13_04234	*0.1*
D -Lactate DH	*dld*	KP13_03193	*−0.1*
L -Lactate DH	*lctD* or *lldD*	KP13_00216	*0.2*
D -Amino acid DH	*dad* (*dad2)*	KP13_04669	**0.9**
Glucose dehydrogenase	*gcD*	KP13_32152	*0.1* ^b^
Succinate DH	*sdhCDAB*	KP13_03281-77	**0.3,** *0.1, 0.1, 0, −0.1*
Quinone biosynthesis:			
Ubiquinone (UQ)	*ubiA*-*ubiH ubiX*	KP13_00377 KP13_01733 KP13_00376 KP13_01739 KP13_01731 KP13_03318 KP13_00956 KP13_02174 KP13_01019	*−0.3* −*0.1* −*0.1* *0.3* *0.1* *0* *0.2* *0.4* *0*
Naphthoquinone menaquinone (MK)	*menB*-*menF menH*	KP13_00975 KP13_00974 KP13_00977 KP13_00973 KP13_00978 KP13_00976	−0.1 0.2 −**0.5** −0.1 0.1 −0.4
Terminal reductases:			
Quinol oxidase bo 3	*cyoABCE*	KP13_03683 KP13_03684 KP13_20485 KP13_03687	*0.1* −*0.1* −*0.2* −*0.1*
Quinol oxidase bd	*cydAB*	KP13_03271-2	**−0.3**
Quinol oxidase III (Cyx)	*appBC*	KP13_04076-5	*0.3*
Nitrate reductase A	*narGHJI*	KP13_04712-09	*0.2,* **0.5**, **0.6**, *0.3*
Nitrate reductase Z	*narZYWV*	KP13_04512-09	**0.6**, *0.3, 0.7, 0.5*
Nitrate reductase, periplasmic	*nasAB*	KP13_04717-8	*0.8*, −*1.1*
DMSO reductase	*dmsBAD*	KP13_01159-61	*0.8, 0.5, −0.2*
Fumarate reductase	*frdABCD*	KP13_31481 KP13_00513 KP13_00512 KP13_00511	*−0.2* −*0.1* **0.5** −**0.3**

Relative expression (log_2_(fold-change) [log_2_FC]) of genes encoding for quinone biosynthesis and oxido-reductases of the respiratory chains of Kp13 for bacterial growth in polymyxin (PB) versus control condition (non-induced). Criteria for significance: FDR≤0.01. log_2_FC values in bold, positive denotes up-regulation in PB and log_2_FC in bold, negative denotes down-regulation in polymyxin. log_2_FC in italic font are not significant based on FDR. ^b^These comparisons gave significant differentially expressed genes at a FDR of ≤0.05

Thus, our results show that polymyxin B affects the expression of several genes encoding for respiratory chain enzymes, which may lead to a shift in alternative respiratory and fermentation pathways for bacterial energy metabolism and to reoxidize the NADH produced by glycolysis. Therefore, we investigated the response of genes encoding for enzymes in these alternative pathways, both by the effect of PB with or without abiotic stimuli. In Additional file 3 we depict a model, according to the data obtained, of carbon flux in Kp13 under fermentative conditions by stresses imposed by PB (Additional file 3, A) or PB plus abiotic stimuli (high calcium/iron, magnesium depletion or low pH) (Additional file 3, B). Consequently to stress imposed by PB without abiotic stimuli the expression of genes encoding for the three enzymatic components of 2-oxoglutarate dehydrogenase complex were slightly repressed (*sucA:* log_2_FC = −0.1 with FDR≤0.05, *sucB:* log_2_FC = −0.3 with FDR≤0.01 and *lpdA*: log_2_FC = −0.2 with FDR≤0.05). This repression was more pronounced with stress of PB in combination with abiotic stimuli, in particular in PB+high Ca (*sucA:* log_2_FC = −1.4, *sucB:* log_2_FC = −0.8 and *lpdA*: log_2_FC = −1.6, all with FDR≤0.01) and PB+low pH (*sucA:* log_2_FC = −2.4, *sucB:* log_2_FC = −1.6 and *lpdA*: log_2_FC = −1.6, all with FDR≤0.01) (Table 3). One explanation for this repression may relate to the observation that *arcA-arcB* showed high expression level following the pressure of PB without abiotic stimuli, and this TCRS represses the expression of the genes involved in the TCA cycle, such as the *sucABCD* system [31]. Under low expression of the genes encoding for the 2-oxoglutarate dehydrogenase complex the citric acid cycle may be interrupted, leading to a shift from aerobic pathways to the fermentation route [31]. Upon PB stress it seems that lactate and acetolactate can be produced actively because *ldhA* and *lvG* showed increased expression following PB treatment, almost independently of abiotic stimuli (Additional file 3, A; Table 3). Conversely, the influence of abiotic changes together with PB induces up-regulation of genes involved in the production of ethanol, acetate and ATP, formate, butane 2,3-ediol and 1,3-propanediol (Additional file 3, B; Table 3). The synthesis of acid by-products would be observable as a lowering in the pH of the medium. To test this hypothesis, we performed an experiment of *K. pneumoniae* Kp13 growth under polymyxin B exposure (analogous to the PB condition in Table 1) and absence, concurrently measuring the medium pH at hourly intervals (see Additional file 4). We observed that, while there is a general trend of acidification of the culture pH, this lowering is more pronounced in bacteria grown under PB stress (Additional file 4), suggesting that PB exposure induces metabolic shifts in *K. pneumoniae* that lead to synthesis of acidic by-products.

The metabolism of glycerol by Kp13 under the pressure of PB (Additional file 3, C) and PB plus abiotic stimuli (Additional file 3, D) were also studied. Notably, it seems that all genes of this pathway showed decreased expression following PB treatment, whereas PB in combination with abiotic stimuli induces up-regulation of all genes for production of 1,3-propanediol.

In this sense, the shift in synthesis of metabolic by-products might reflect a decrease in NADH oxidation via the respiratory chain, which is compensated by the NADH consuming lactate or acetolactate (under PB stress), and ethanol or 1,3-propanediol (under PB+abiotic stimuli stresses) production.

On the other hand, disruption in the electron transport chain induces the production of superoxide (HO_2_^.-^) that is converted in hydrogen peroxide (H_2_O_2_), which in turn participate in the Fenton reaction and additionally induce synthesis of hydroxyl radicals [38, 39]. Large concentrations of superoxide and hydrogen peroxide trigger bacterial antioxidant defenses, but small amounts of hydroxyl radicals (OH^−^) do not elicit adequate defense mechanisms and could quickly become fatal [38, 40]. In Gram-negative bacteria, the intracellular superoxide dismutases Mn-SodA and Fe-SodB, catalyze the dismutation of metabolic sources of superoxide to hydrogen peroxide and oxygen [41]. Then, in the presence of transition iron ions (Fe^2+^), the hydrogen peroxide can be converted to hydroxyl radicals via Fenton chemistry: Fe^2+^ + H_2_O_2_ → Fe^3+^ + OH^.^ + OH^−^. In bacteria, the scavenging enzymes that can resolve the hydrogen peroxide production are glutathione peroxidases and catalases [39, 40]. For that, glutathione peroxidases by oxidation of glutathione (GSH): 2GSH + H_2_O_2_ → GSSG + 2H_2_O and catalase via the reaction 2H_2_O_2_ → O_2_ + 2H_2_O [42].

In our RNA-seq analysis, we observed that *sodA* (KP13_00587) was up-regulated in PB without the influence of abiotic changes, whereas *sodB* (KP13_05143) appeared slightly overexpressed in high iron and magnesium deprivation conditions (Additional file 2). Genes encoding for a glutathione peroxidase (KP13_31955) and Catalase HPII (*katE,* KP13_05384) were overexpressed in polymyxin B independently of abiotic changes. While *katG* (KP13_04579) encoding for a catalase-peroxidase bufunctional enzyme appeared overexpressed in high calcium/iron conditions (Additional file 2).

One of the mechanisms used by bacteria to cope with the oxidative stress induced by Fenton products involve the intracellular regulation of iron metabolism mediated by bacterioferritin (Bfr) or ferritin-like proteins. These proteins sequester intracellular Fe^2+^ ions and stores it in the form of Fe^3+^, where one hydrogen peroxide oxidizes two Fe^2+^ ions, thus preventing hydroxyl radical production by the Fenton reaction [43]. Here, we identified a gene encoding for ferritin-like protein (KP13_13049) overexpressed in PB considering all abiotic changes, with a specially consistent up-regulation in PB with high iron condition (log_2_FC = 1.6) (Additional file 2). It has been reported that the production of hydroxyl radicals induced by polymyxin treatment is concurrent with rapid killing of Gram-negative *A. baumannii* [36].

Additionally, Fenton oxidants cause oxidation of DNA and free nucleotides, which can induce mismatched pairing, mutagenesis, and DNA breaks. We detected significant elevated relative expression of genes encoding for DNA damage repair proteins in polymyxin B with acid pH (*recF* - KP13_00050 - log_2_FC = 2.8; *recO* - KP13_00815 - log_2_FC = 0.7, *recR -* KP13_03619 - log_2_FC = 2.3; *uvrA* - KP13_00395 - log_2_FC = 2.3; *uvrB* - KP13_03032 - log_2_FC = 0.2; *uvrC* - KP13_01656 - log_2_FC = 1.2) and high iron condition (*recN* - KP13_02431 - log_2_FC = 0.2) (see Additional file 2). These observations were confirmed by the detection of co-expressed gene modules related to DNA damage repair processes, which are presented in the last section of the results.

Also, we identified that gene expression level of *tisB* (KP13_31738, Additional file 2), which codes a protein part of the SOS-response regulon controlled by LexA, was increased during PB pressure with high calcium/iron condition (log_2_FC>1.4) (Additional file 2). In *E. coli*, it has been shown that this protein is toxic since its overproduction cause slow or stop bacterial growth, probably allowing DNA repair before cells continue to grow [44].

With regard to the defense mechanisms against acidic pH conditions, we identified a gene (KP13_00169) encoding for a chloride channel/voltage gated protein up-regulated in PB with all abiotic changes (log_2_FC>1.4), but having considerable higher overexpression in acid pH condition (log_2_FC = 3.5) (Additional file 2). This protein belongs to the chloride channel (TC 2.A.49) family and probably acts as an electrical shunt for an outwardly-directed proton pump that is linked to amino acid decarboxylation, as part of the acid resistance response [45].

On the other hand, we also identified PB-dependent overexpression of a few genes encoding for proteins that are activated in response to cold shock, such as *deaD* (KP13_01093, cold-shock DEAD box protein A with highest log_2_FC>2.9 in PB with acid pH condition), *cspA* (KP13_00250, cold shock protein CspA with highest log_2_FC>4.8 in PB with acid pH condition) (Additional file 2).

We performed quantitative real-time PCR (qRT-PCR) to independently validate the expression of some of the studied genes (*phoP*, *pmrB* and *arcB*). These genes were chosen based on their involvement in the response to PB exposure, as discussed, for instance, with *arcB.* With few exceptions, the three genes displayed similar expression trends in the various experimental conditions tested, thus validating the digital gene expression measurements provided by the RNA-seq experiments (Additional file 5).

### Environmental stimuli elicit differential responses in the expression of regulatory systems

**Fig. 2:**
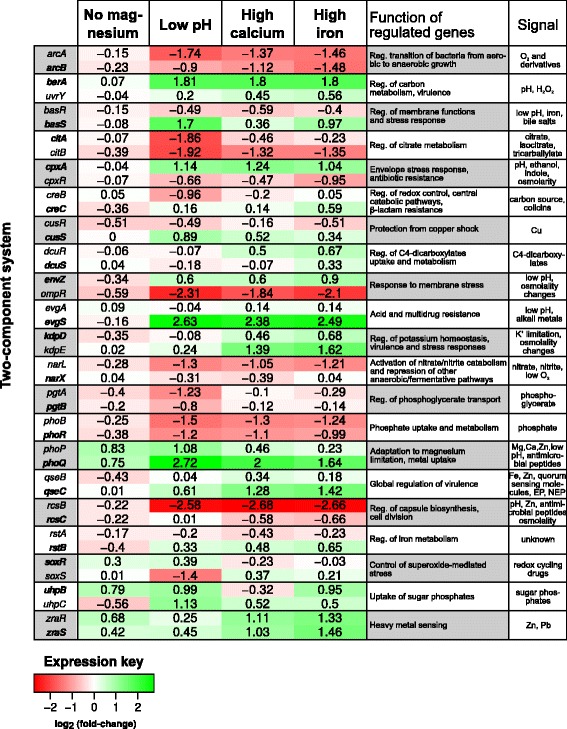
Expression of identified two-component systems in *K. pneumoniae* Kp13 with distinct environmental stimuli. Expression values are represented as log_2_(fold-change) of the condition compared to the PB condition (log_2_(*abiotic condition*/*PB condition*). Thus, a positive log_2_FC value indicates higher expression of the gene in face of abiotic stress to which it was subjected. For each system, the sensor protein is highlighted in *bold*, while the regulatory component is in normal formatting. The main functions and signals perceived by each system are shown according to literature mining

We have also studied the expression of other identified TCRS. While magnesium deprivation generated low-level repression on most of these regulatory circuits, in acid pH and high calcium/iron conditions, a similar pattern of activation/repression of regulatory genes was observed, leading to their clustering (Additional file 6). For instance, genes *arcA, arcB, phoB, phoR, narL, citB, ompR* and *rcsB* showed repression (log_2_FC < −1.0), considering the abiotic condition relative to PB condition (Fig. 2). The expression of *arcB* was validated by qRT-PCR, as previously observed. PhoBR has been primarily implicated in phosphate homeostasis, and its involvement in stress responses such as acidic pH was previously reported [46]. That this system appears repressed when considering only the influence of abiotic changes suggests the participation of other regulatory mechanisms or signals controlling its expression in *K. pneumoniae. narL* and *citB* are involved in anaerobic nitrate and citrate metabolism [47, 48], and were also found repressed, specially when the bacteria are grown in acidic medium, with log_2_FC of −1.3 (*narL*) and −1.92 (*citB*). The metabolic shift from aerobic to anaerobic in response to PB, through alternative pathways, was previously explored in this report. *ompR* is part of a TCRS along with *envZ* that responds to external osmolarity, while *rcsB* is one of the mediators of capsule synthesis [49].

On the other hand, differential activation of several regulators in specific abiotic stimuli were detected in our experiments, including the *phoP-phoQ* system which was found more activated in low pH and high calcium/iron conditions (Fig. 2; Additional file 5); *zraR-zraS* is a zinc-responsive TCRS involved in tolerance to metals and its differential activation occurred mostly in high calcium and iron conditions. This system has been regarded as important for pathogenic *Salmonella* during infection in pig [50]. *basR* (or *pmrA*) and *basS* (or *pmrB*), which have been implicated in PB resistance, appear overexpressed in low pH and high iron conditions (Fig. 2), in line with previous observations [51]. In high iron condition, however, there were opposite responses comparing the RNA-seq data with the qRT-PCR, where in the latter *pmrB* appeared repressed. *cpxA*, part of the CpxA-CpxR TCRS, appeared slightly overexpressed in acid pH and high iron/calcium concentrations (Fig. 2). This system is involved in envelope stress response, adhesion, motility and pathogenesis, and modulates the expression of a large number of genes [30]. Furthermore, *cpxA-cpxR* has also been implicated in oxidative stress tolerance in *K. pneumoniae* [52].

We have also identified a strong (log_2_FC≥2) activation in acid pH and high calcium/iron conditions of *evgS*, which form part of a TCRS along with *evgA*. EvgA-EvgS system has been reported as modulator of the expression of several genes including multidrug-related, mostly in response to osmolarity changes in the medium. Thus, the finding that the sensor component *evgS* was more activated in the low pH condition was not unexpected. The analysis of genes previously reported to be under regulation of this system in *E. coli* [53] allowed the identification of orthologs in *K. pneumoniae* Kp13 that display similar expression response in the low pH condition. For instance, we identified in Kp13 an ortholog of *yfdX* (55 % identity to the *E. coli* sequence, BLASTP; Kp13 locus KP13_31877), that is strongly activated in low pH condition (log_2_FC = 2.0), in a similar fashion to the cognate gene in *E. coli* [53]. These collateral results are of value to better explore the uncharacterized fraction of *K. pneumoniae* genes and represent a complementary result attained by this transcriptomic study in better delineating conserved regulatory responses in clinically important bacteria.

### Co-expression network analysis reveals functional signatures related to polymyxin B, iron limitation and acidic pH

**Fig. 3:**
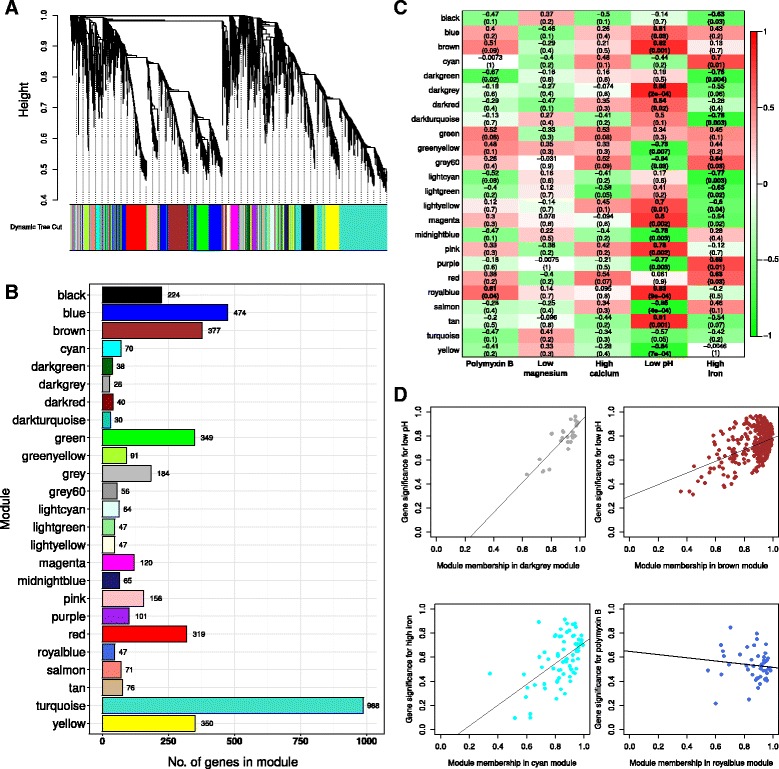
Determination of co-expressed gene modules related to PB and abiotic stresses. Modules were identified using hierarchical clustering followed by dynamic tree cutting algorithm in WGCNA. Each co-expression module was assigned to a unique color (Panel **a**). The number of genes that grouped into each module ranged from 28 (module *darkgrey*) to 988 (module *turquoise*) (Panel **b**). Modules were related to our experimental conditions, and we could detect modules significantly correlated to polymyxin B, low pH and high iron (marked in *bold* in Panel **c**). The membership of each gene to its module and its significance to the experimental condition is plotted for selected modules in Panel **d**. These genes can be regarded as key drivers in these modules

To determine gene-expression signatures related to abiotic stresses and PB treatment, we performed co-expression network analysis using the variance-stabilized count data as input. The premise of this analysis is that co-expressed genes are biologically related, and thus can be grouped into clusters or modules. The modules were differentiated by assigning to each a color label, with *grey* reserved for those that remained unassigned due to low co-expression. A total of 24 modules were detected using this approach, with the smaller module comprising 28 genes (module *darkgrey*) and the largest module having 988 genes (module *turquoise*) (Fig. 3a and b). We calculated module eigengene, which is the first principal component of each module, summarizing the within-module expression pattern, and used it to assess whether modules related to our RNA-seq experimental conditions. Some of the modules displayed significant correlation (marked in bold in Fig. 3c) and might shed further light on the more fine-grained molecular aspects involved in the bacterial response to the different stimuli in our experiments. For instance, module *darkgrey* had module eigengene positively correlated to the acid medium condition (Pearson’s *r* = 0.88; *p*-value *=* 2 × 10^−4^) (Fig. 3c); that is, genes in this module displayed expression patterns that were also associated to the low pH condition (Fig. 3d). The *brown* module was also positively associated to low pH (Pearson’s *r* = 0.82; *p*-value *=* 1 × 10^−3^) (Fig. 3d), while modules *cyan* and *royalblue* could be related to high iron and PB exposure, respectively (Fig. 3c and d). Some modules were also anti-correlated to the conditions, such as module *yellow* which was negatively correlated to acid pH condition (Pearson’s *r =* 0.84; *p*-value *=* 7 × 10^−4^). In order to understand their biological relevance, these significant modules were studied in detail by means of Gene Ontology enrichment analysis. Modules and associated genes and GO terms are available in Additional file 7.

**Fig. 4:**
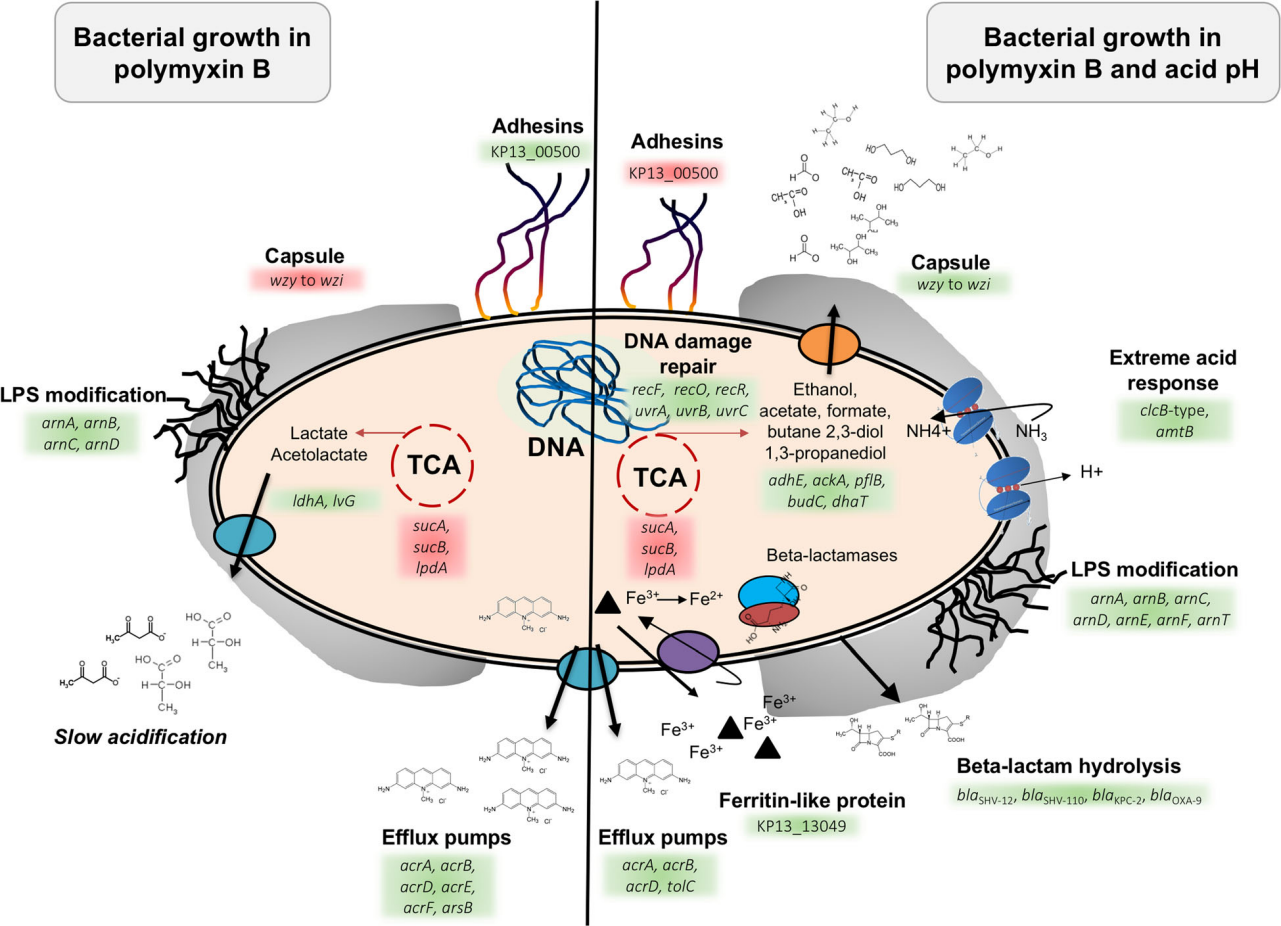
Diagram showing the response of *K. pneumoniae* Kp13 to PB and PB with acid pH. High polymyxin B concentration (*left side*) and high polymyxin B concentration with acid pH (*right side*). The mechanisms involved in Kp13’s response in both culture conditions are described in this manuscript. These mechanisms were ranging from LPS modification, capsule and adhesion production, metabolic shift aerobic to fermentation, efflux pump and beta-lactamases overexpression, regulation of iron metabolism (ferritin-like protein), acid response and DNA damage repair

Module *darkgrey* comprised 28 genes with associated GO terms related to glutamate biosynthetic process (Benjamini-Hochberg [BH] adjusted *p*-value *=* 0.1), ATP hydrolysis coupled proton transport (BH-adjusted *p*-value *=* 0.1) and protein metabolic processes such as protein complex disassembly (BH-adjusted *p*-value *=* 0.01). The finding that this module is strongly, positively correlated to the low pH condition relates to described mechanisms of acid tolerance in bacteria. Although terms related to glutamate synthesis had low statistical significance, probably due to the small module size, their identification confirms literature evidence that glutamate is important for bacterial survival in low pH, as it can be decarboxylated by specific enzymes which consume proton in the process [54]. Also, proton transport across the membrane by means of ATP synthase is an active strategy to maintain pH homeostasis on the cell interior, pumping excess H^+^ out of the cell. The gene encoding for subunit alpha of ATP synthase (*atpA*) was placed in this module, and its expression was slightly up-regulated in low pH when compared to the control PB condition grown at pH 7.0 (log_2_FC = 0.6). In fact, all subunits of ATP synthase are more activated when in acid pH, indication of a consistent pattern and involvement of this enzyme in response to acid stress (Additional file 2), as has been reported for other bacteria [55]. The *pink* module, which also displayed positive correlation to the acid pH condition (Fig. 3), included ATP synthase genes *atpG, atpD* and *atpC*, involved in ATP synthesis coupled proton transport (BH-adjusted *p*-value *=* 7.5 × 10^−3^). The log_2_FC of these genes relative to the PB condition were of 2.00, 1.97 and 2.97, respectively. The presence of this biological process in multiple modules related to low pH might punctuate a role in face of acid stress. Terms related to ribosome biogenesis (BH-adjusted *p*-value *=* 0.01) and RNA processing (BH-adjusted *p*-value *=* 0.01) were also enriched in this module, as well as branched-chain amino acid (BCAA) biosynthetic process (BH-adjusted *p*-value *=* 0.03), with genes such as *ilvC, leuB* and *leuC* forming part of the module. Routing of metabolism to produce BCAA is a mechanism employed by bacteria to survive acid stress, as they can be further decarboxylated by proton-consuming enzymes [56], which were previously identified in module *darkgrey*. Also in module *pink* were genes *acrA* and *acrB* coding for an acriflavine efflux pump. It is known that *K. pneumoniae* uses the AcrAB-TolC efflux pump system to mediate resistance against fluoroquinolones and to resist CAMPs [57]. Also, in our experiments, the genes *acrA*, *acrB* and *tolC* were more expressed in PB with acid pH (Additional files 2 and 5; also see scheme in Fig. 4).

Module *brown*, also related to low pH, was enriched in terms related to ion transmembrane transport (BH-adjusted *p*-value *=* 0.2), which includes genes *amtB* (encoding for an ammonia channel) and the chloride channel KP13_00169 (previously discussed), mechanisms which would allow, respectively, for the entrance of NH_3_ (that can react with H^+^ yielding NH_4_^+^ and increasing pH) and the pumping out of H^+^ species [56]. Indeed, *amtB* expression is higher when in low pH condition (log_2_FC = 1.1). In this module was also found *arnB, arnC, arnD, arnE, arnF* and *arnT* genes, encoding for proteins that participate in the addition of 4-amino-4-deoxy-L-arabinose to lipid A, contributing to a more positive charge on LPS, which in turn decreases PB affinity to the membrane [58]. ArnA, encoded by gene *arnA* (found in the *pink* module), is also involved in this process, and was strongly up-regulated in acid pH (log_2_FC = 3.1; low pH vs. PB condition). The *arn* genes showed highest overexpression in PB with low pH. Genes related to stress and cold shock response (*cspA, deaD*), previously discussed, were found up-regulated in acid pH and also belong to this module.

The *yellow* module, having 350 associated genes, presented negative correlation to the low pH condition (Pearson’s *r = −*0.84; *p*-value *=* 7 × 10^−4^). Overall, it appears that this module comprises genes mostly related to respiration and oxidative cell processes. Functional categories identified in this module included aerobic respiration (BH-adjusted *p*-value *=* 3.7 × 10^−5^; genes *sdhA, sdhB, sdhC, sdhD* encoding for succinate DH). These genes were previously discussed in the context of PB presence, appearing slightly activated in PB exposure. When considering acid stress, however, these genes were strongly down-regulated, with log_2_FC values between −2.9 (*sdhC*) and −3.5 (*shdA*) (comparing low pH to PB). Generation of precursor metabolites and energy (BH-adjusted *p*-value *=* 2.1 × 10^−5^) was also a significantly enriched term, and genes encoding for ubiquinol oxidase (*cyoAB*), components of 2-oxoglutarate dehydrogenase complex (*sucAB*)*,* succinyl-CoA ligase (*sucC*) could be related to these metabolic processes. All of these genes were found down-regulated when in acid pH. Also present were small heat shock proteins coded in the *ibpA*/*ibpB* genes as well as universal stress proteins A/B (*uspA/B*). *uspA* is reported to be highly transcribed in response to growth stasis, starvation or stress agents [59]. Down-regulation of *uspA* (log_2_FC = −1.8) and *uspB* (log_2_FC = −3.5) in acid pH were identified in our analyses. The combined evidence argues for a diminished oxidative activity combined with stress response factors when in acid pH.

Another module of interest was the *tan*, which contains 76 genes with associated functional terms including response to antibiotic (BH-adjusted *p*-value *=* 0.002), DNA repair (BH-adjusted *p*-value *=* 0.08), DNA topological change (BH-adjusted *p*-value *=* 0.02). The involved genes include *uvrB, dnaB, gyrA, parC, macA.* Also, beta-lactamases *bla*_SHV-12_ and *bla*_SHV-110_ and genes encoding for fimbrial protein (KP13_04093), chaperone genes (*hscAB*) were found in this module. Although not directly related to PB resistance, it is interesting to note the activation of *bla* genes in PB with acid pH. While module *tan* correlated strongly only to the acid condition (Fig. 4), the finding that it groups resistance, virulence and DNA repair genes warrants further study on the common regulatory mechanisms that might control their expression, as disruption of these genes would affect many important correlates of infection and resistance.

Module *darkgreen,* which groups 38 genes, comprised several GO terms related to translation (adjusted *p*-value *=* 2.2 × 10^−13^) and peptide metabolic processses (adjusted *p*-value *=* 1.4 × 10^−9^); genes involved in this module included elongation factor (*fusA*, KP13_00742), several ribosomal subunit proteins (*rplA*, *rplC, rplD, rplX, rplR, rplE, rplO, rplI, rplN, rplL, rpsA*) and a protein translocase (*secA,* KP13_01897). That these ribosomal genes group into the same module is consistent with previous reports indicating their cotranscription in other bacteria [60], thus supporting the co-expression based analytical approach that we undertook. The negative correlation of this module with high iron and PB exposure hints an effect that protein synthesis might be inhibited at these conditions. Most interestingly is the possibility that polymyxin B could be exherting an inhibitory activity within the cell interior and effectively blocking these cellular processes, an observation that is in line with literature evidence for other CAMPs [61, 62]. This is also in agreement with our data that PB not only disrupts membrane functions, but also targets several central cellular processes such as carbon metabolic pathways (previously discussed) and provides further evidence to the idea that membrane disruption per se does not justify the killing of bacterial cells by these drugs, which probably involves additional intracellular targets.

It is worth noting that the genes coding for capsule biosynthesis (*cps*_Kp13_, which we previously studied in [14]) did not cluster in any single module. This could be because capsule production involves diverse biological processes, ranging from oligosaccharide chain biosynthesis, the polymerization of this chain and transport through the inner to the external membrane [63]. As such, each of these steps should be regulated independently, hindering the clustering of these genes based in co-expression patterns alone. However, there are several evidences that capsule overproduction is also correlated with resistance to polymyxins [64, 65]. This phenomenon was observed in our RNA-seq experiments, but was only consistent for almost the whole *cps*_Kp13_ gene cluster in PB with low pH condition (Additional file 2 and Fig. 4).

The study of co-expressed modules and their association to the different experimental conditions shed light on important aspects underlying the *K. pneumoniae* response to PB and to abiotic stresses. Our results are consistent with the proposition that multiple metabolic aspects are affected in the course of PB exposure, and bacteria display a coordinated response which involves shifts in oxidative mechanisms, respiratory control, amino acid usage and transformation as well as regulatory components.

## Conclusions

The present report provides a comprehensive gene expression dataset related to polymyxin B treatment under diverse abiotic stresses using *K. pneumoniae* Kp13 as model. We emphasize the functional transcriptomic aspects which are governed by PB treatment and the regulatory responses that may relate to exposure and resistance to this antibiotic, pinpointing intracellular targets that appear linked to the response elicited by PB exposure, such as the ArcA-ArcB system, previously unlinked to polymyxin response. Here, we highlight that polymyxin B exposure induced a metabolic shift into fermentative growth in *Klebsiella pneumoniae* Kp13.

Since PB resistance is an increasing trend in MDR bacteria, the study of its emergence by combination of expression profiling data from diverse bacteria, expanding on the present dataset, may aid on the search for common traits of resistance mechanisms linked to PB. A better understanding of these mechanisms will support the search of alternative or combination therapies to control these hard-to-treat infections.

## Additional files

Additional file 1: Genes with a shared PB-dependent differential expression pattern (core PB treatment independent of abiotic stresses). (DOC 101 kb)
Additional file 2: Differentially expressed genes in polymyxin in comparison with five environmental conditions. (XLS 1099 kb)
Additional file 3: Scheme of carbon flux in *Klebsiella pneumoniae* Kp13 under different abiotic conditions and polymyxin B exposure. Description: A) Scheme of carbon flux in *Klebsiella pneumoniae* Kp13 under fermentative conditions induced by stresses imposed by PB; B) Scheme of carbon flux in *Klebsiella pneumoniae* Kp13 under fermentative conditions induced by stresses imposed by PB plus abiotic stimuli (High Ca, High Fe, Mg deprivation or Low pH); C) The metabolism of glycerol by *Klebsiella pneumoniae* under the pressure of PB; D) The metabolism of glycerol by *Klebsiella pneumoniae* under the pressure of PB plus abiotic stimuli (High Ca, High Fe, Mg deprivation or Low pH). (PDF 145 kb)
Additional file 4:
*Klebsiella pneumoniae* Kp13 growth under polymyxin B (PB) exposure vs. non-induced bacteria grown without PB. Description: Bacterial growth and medium pH were recorded at hourly intervals at seven timepoints. (PDF 46 kb)
Additional file 5: Validation of the expression of *pmrB, arcA* and *phoB* using qRT-PCR. Description: The log_2_-transformed expression values of each gene, relative to the PB condition (strain Kp13_PolB_), is reported for both the RNA-seq data and qRT-PCR measurements. (PDF 40 kb)
Additional file 6: Expression heatmap of regulatory systems. Description: Expression values are represented as log2(fold-change) of the condition compared to the PB condition (log2(*abiotic condition/PB condition*). Thus, a positive log_2_FC value indicates higher expression of the gene in face of abiotic stress to which it was subjected. In this figure, all genes that we identified as a possible two-component regulatory system (by sequence similarity, conserved domains, genomic positional evidence and co-occurrence) are depicted. Genes for which we failed to identify a known homolog in the literature and databases are only cited by their locus tag in Kp13 strain. (PDF 79 kb)
Additional file 7: Modules of co-expressed genes as revealed by weighted gene correlation network analysis. Description: Reported are the modules (labeled by different colours) to which each gene belongs, as well as the associated Gene Ontology terms. (XLS 734 kb)
