# Supplementary material for: The polymyxin B-induced transcriptomic response of a clinical, multidrug-resistant Klebsiella pneumoniae involves multiple regulatory elements and intracellular targets
Source: BMC Genomics. 2016 Oct 25;17(Suppl 8):737. doi: 10.1186/s12864-016-3070-y (PMC5088521; doi:10.1186/s12864-016-3070-y)
Supplement: Additional file 1: — Genes with a shared PB-dependent differential expression pattern (core PB treatment independent of abiotic stresses). (DOC 101 kb) [file 12864_2016_3070_MOESM1_ESM.doc]

**Additional File 1 -** Genes with a shared PB-dependent differential expression pattern (core PB treatment independent of abiotic stresses). Note that, for this analysis, the PB condition (Kp13_PolB_ strain) was used as test and every other condition in the RNA-seq experiment was used as reference.

| **Locus ID** | **Gene name** | **Replicon** | **Product name** | **log_2_FC(PB/Condition)** | | | | |
| --- | --- | --- | --- | --- | --- | --- | --- | --- |
|  |  |  |  | **No PB** | **Calcium** | **Iron** | **Magnesium** | **pH** |
| **Up-regulated** | | | |  |  |  |  |  |
| KP13_04991 | *astC* | KP13_chr | Succinylornithine transaminase | 1.83 | 4.57 | 2.72 | 1.55 | 6.07 |
| KP13_04994 | *astB* | KP13_chr | N-succinylarginine dihydrolase | 1.65 | 4.70 | 2.20 | 2.12 | 5.65 |
| KP13_00030 | *pstS* | KP13_chr | Phosphate-binding protein pstS | 0.30 | 5.06 | 4.94 | 0.54 | 4.24 |
| KP13_09592 | *-* | KP13_chr | Leucine-specific-binding protein | 0.88 | 2.82 | 2.54 | 0.93 | 4.02 |
| KP13_03923 | *putP* | KP13_chr | Sodium/proline symporter | 0.53 | 2.58 | 2.41 | 0.72 | 4.01 |
| KP13_04725 | *livJ* | KP13_chr | Leu/Ile/Val-binding protein | 0.46 | 2.44 | 2.44 | 0.76 | 3.92 |
| KP13_03524 | *-* | KP13_chr | putative periplasmic binding protein/LacI transcriptional regulator | 1.06 | 2.46 | 1.79 | 0.44 | 3.81 |
| KP13_00225 | *-* | KP13_chr | Inner membrane protein | 0.69 | 3.15 | 2.51 | 0.73 | 3.77 |
| KP13_00647 | *ugpB* | KP13_chr | sn-glycerol-3-phosphate-binding periplasmic protein ugpB | 0.89 | 2.79 | 2.37 | 0.91 | 3.61 |
| KP13_00433 | *acs* | KP13_chr | Acetyl-coenzyme A synthetase | 0.61 | 1.33 | 0.64 | 1.04 | 3.59 |
| KP13_00312 | *uspB* | KP13_chr | Universal stress protein B | 0.37 | 2.29 | 2.01 | 0.74 | 3.52 |
| KP13_02931 | *fadE* | KP13_chr | Acyl-coenzyme A dehydrogenase | 0.27 | 1.37 | 0.96 | 0.53 | 3.46 |
| KP13_00218 | *lldP* | KP13_chr | L-lactate permease | 0.96 | 1.68 | 0.90 | 0.93 | 3.41 |
| KP13_31540 | *asnA* | KP13_chr | Aspartate--ammonia ligase | 0.46 | 2.95 | 3.29 | 0.69 | 2.42 |
| KP13_05398 | *astA* | KP13_chr | Arginine N-succinyltransferase | 0.71 | 2.04 | 1.35 | 1.05 | 3.11 |
| KP13_00653 | *ggt* | KP13_chr | Gamma-glutamyltranspeptidase | 0.60 | 1.88 | 1.27 | 1.11 | 2.70 |
| KP13_03016 | *-* | KP13_chr | putative ATPase, P-type | 0.42 | 1.52 | 0.95 | 0.28 | 2.69 |
| KP13_00936 | *nudK* | KP13_chr | GDP-mannose pyrophosphatase nudK | 1.52 | 1.53 | 1.61 | 1.14 | 2.66 |
| KP13_03008 | *-* | KP13_chr | putative HTH-type transcriptional regulator | 0.80 | 1.82 | 1.97 | 0.96 | 2.36 |
| KP13_00688 | *ompR* | KP13_chr | Transcriptional regulatory protein ompR | 0.46 | 1.84 | 2.10 | 0.59 | 2.31 |
| KP13_00736 | *-* | KP13_chr | hypothetical protein | 0.39 | 1.76 | 2.04 | 0.34 | 0.51 |
| KP13_03999 | *degQ* | KP13_chr | Protease degQ | 0.25 | 1.33 | 1.26 | 0.79 | 1.99 |
| KP13_01107 | *secG* | KP13_chr | Protein-export membrane protein secG | 0.80 | 1.66 | 1.96 | 0.49 | 1.55 |
| KP13_03006 | *-* | KP13_chr | Blue light- and temperature-regulated antirepressor | 0.73 | 1.59 | 1.39 | 0.40 | 1.95 |
| KP13_01097 | *rpsO* | KP13_chr | 30S ribosomal protein S15 | 0.18 | 1.11 | 1.90 | 0.37 | 0.45 |
| KP13_00662 | *asd* | KP13_chr | Aspartate-semialdehyde dehydrogenase | 0.38 | 1.78 | 1.75 | 0.89 | 1.26 |
| KP13_00251 | *-* | KP13_chr | putative HTH-type transcriptional regulator | 0.40 | 1.23 | 1.38 | 0.29 | 1.57 |
| KP13_03225 | *arcB* | KP13_chr | Aerobic respiration control sensor protein arcB | 0.51 | 1.12 | 1.48 | 0.23 | 0.90 |
| KP13_31778 | *argR* | KP13_chr | arginine repressor | 0.38 | 1.46 | 1.47 | 0.43 | 0.99 |
| KP13_05167 | *tppB* | KP13_chr | Tripeptide permease tppB | 0.25 | 1.43 | 1.36 | 0.29 | 1.24 |
| KP13_01230 | *cycA* | KP13_chr | D-serine/D-alanine/glycine transporter | 0.84 | 1.26 | 1.04 | 0.69 | 1.38 |
| KP13_01115 | *obg* | KP13_chr | GTPase obg | 0.28 | 0.94 | 1.21 | 0.39 | 0.35 |
| KP13_08477 | *rpoH* | KP13_chr | RNA polymerase sigma-32 factor | 0.60 | 1.06 | 1.20 | 0.55 | 0.40 |
| KP13_05000 | *aldA* | KP13_chr | Lactaldehyde dehydrogenase | 0.44 | 1.14 | 1.09 | 0.57 | 1.11 |
| KP13_00684 | *feoB* | KP13_chr | Ferrous iron transport protein B | 0.24 | 1.02 | 0.52 | 0.62 | 1.10 |
| KP13_01110 | *ftsH* | KP13_chr | Cell division protease ftsH | 0.47 | 0.71 | 1.00 | 0.42 | 0.35 |
| KP13_00679 | *nfuA* | KP13_chr | Fe/S biogenesis protein nfuA | 0.98 | 0.86 | 0.99 | 0.67 | 0.37 |
| KP13_00313 | *pitA* | KP13_chr | Low-affinity inorganic phosphate transporter 1 | 0.53 | 0.84 | 0.62 | 0.51 | 0.97 |
| KP13_00342 | *ftsE* | KP13_chr | Cell division ATP-binding protein ftsE | 0.57 | 0.70 | 0.86 | 0.72 | 0.31 |
| KP13_13130 | *wzzE* | KP13_chr | Lipopolysaccharide biosynthesis protein wzzE | 0.34 | 0.74 | 0.54 | 0.34 | 0.33 |
| KP13_03232 | *rpoN* | KP13_chr | RNA polymerase sigma-54 factor | 0.20 | 0.42 | 0.52 | 0.50 | 0.32 |
| **Down-regulated** | | | |  |  |  |  |  |
| KP13_00963 | *glpB* | KP13_chr | Anaerobic glycerol-3-phosphate dehydrogenase subunit B | -2.05 | -6.72 | -7.38 | -2.40 | -5.28 |
| KP13_00962 | *glpA* | KP13_chr | Anaerobic glycerol-3-phosphate dehydrogenase subunit A | -3.56 | -6.99 | -7.30 | -3.47 | -6.00 |
| KP13_00964 | *glpC* | KP13_chr | Anaerobic glycerol-3-phosphate dehydrogenase subunit C | -1.38 | -6.45 | -6.92 | -2.22 | -4.88 |
| KP13_02850 | *dhaM* | KP13_chr | PTS-dependent dihydroxyacetone kinase, phosphotransferase subunit dhaM | -1.23 | -5.33 | -5.32 | -0.42 | -3.39 |
| KP13_02845 | *dhaD* | KP13_chr | Glycerol dehydrogenase | -0.84 | -5.25 | -5.02 | -0.75 | -3.31 |
| KP13_02848 | *dhaK* | KP13_chr | PTS-dependent dihydroxyacetone kinase, dihydroxyacetone-binding subunit dhaK | -1.18 | -5.15 | -4.98 | -0.37 | -3.24 |
| KP13_32382 | - | KP13_chr | hypothetical protein | -0.63 | -4.06 | -4.82 | -0.79 | -2.17 |
| KP13_00961 | *glpT* | KP13_chr | Glycerol-3-phosphate transporter | -1.79 | -3.17 | -3.35 | -1.92 | -1.41 |
| KP13_31830 | *mrkA* | KP13_chr | Type 3 fimbrial subunit MrkA | -2.18 | -2.95 | -2.16 | -0.39 | -3.17 |
| KP13_31826 | *murC* | KP13_chr | UDP-N-acetylmuramate--L-alanine ligase | -0.53 | -2.98 | -3.15 | -0.39 | -3.00 |
| KP13_31552 | *glpF* | KP13_chr | Glycerol uptake facilitator protein | -1.73 | -2.77 | -3.07 | -1.65 | -0.70 |
| KP13_00026 | *atpC* | KP13_chr | ATP synthase epsilon chain | -0.37 | -2.44 | -1.57 | -0.37 | -2.97 |
| KP13_00960 | *glpQ* | KP13_chr | Glycerophosphoryl diester phosphodiesterase | -0.98 | -2.55 | -2.66 | -1.39 | -0.81 |
| KP13_00757 | *rpmC* | KP13_chr | 50S ribosomal protein L29 | -1.13 | -2.55 | -1.55 | -0.96 | -2.28 |
| KP13_01285 | *treB* | KP13_chr | PTS system trehalose-specific EIIBC component | -0.41 | -1.56 | -0.86 | -0.24 | -2.44 |
| KP13_31628 | *rimM* | KP13_chr | Ribosome maturation factor rimM | -0.84 | -1.69 | -0.87 | -0.43 | -2.28 |
| KP13_00759 | - | KP13_chr | hypothetical protein | -1.08 | -2.01 | -1.08 | -0.65 | -1.71 |
| KP13_01362 | *dcuB* | KP13_chr | Anaerobic C4-dicarboxylate transporter dcuB | -0.56 | -1.98 | -1.79 | -0.25 | -1.53 |
| KP13_00670 | *glpD* | KP13_chr | Aerobic glycerol-3-phosphate dehydrogenase | -1.50 | -1.73 | -1.95 | -1.41 | -0.61 |
| KP13_02423 | *trmD* | KP13_chr | tRNA (guanine-N(1)-)-methyltransferase | -0.79 | -1.23 | -0.47 | -0.37 | -1.81 |
| KP13_05370 | - | KP13_chr | PKHD-type hydroxylase | -0.57 | -1.73 | -1.60 | -0.62 | -0.60 |
| KP13_02372 | - | KP13_chr | LOG family protein | -0.22 | -0.83 | -0.73 | -0.69 | -1.67 |
| KP13_02811 | *plsY* | KP13_chr | Glycerol-3-phosphate acyltransferase | -0.69 | -1.43 | -1.44 | -0.75 | -0.87 |
| KP13_01225 | *rpsR* | KP13_chr | 30S ribosomal protein S18 | -1.03 | -1.32 | -0.81 | -0.56 | -1.15 |
| KP13_06886 | - | PKP13d | conjugal transfer TrbC-like protein | -1.27 | -1.32 | -1.27 | -0.66 | -0.68 |
| KP13_01223 | *rpsF* | KP13_chr | 30S ribosomal protein S6 | -0.75 | -1.32 | -0.63 | -0.33 | -0.99 |
| KP13_02868 | - | KP13_chr | putative oxidoreductase | -0.37 | -1.26 | -1.04 | -0.44 | -1.07 |
| KP13_06748 | *topB* | PKP13d | DNA topoisomerase III | -0.34 | -1.21 | -1.16 | -0.46 | -0.38 |

Only genes with FDR≤0.01 are shown. Fold-change values for each condition are shown as the log_2_-transformed proportion of the PB condition relative to the compared condition. The table is sorted according to the maximum (up-regulated genes) or minimum (down-regulated genes) fold-change value at any condition.
