# Supplementary material for: The polymyxin B-induced transcriptomic response of a clinical, multidrug-resistant Klebsiella pneumoniae involves multiple regulatory elements and intracellular targets
Source: BMC Genomics. 2016 Oct 25;17(Suppl 8):737. doi: 10.1186/s12864-016-3070-y (PMC5088521; doi:10.1186/s12864-016-3070-y)
Supplement: Additional file 3: — Scheme of carbon flux in Klebsiella pneumoniae Kp13 under different abiotic conditions and polymyxin B exposure. Description: A) Scheme of carbon flux in Klebsiella pneumoniae Kp13 under fermentative conditions induced by stresses imposed by PB; B) Scheme of carbon flux in Klebsiella pneumoniae Kp13 under fermentative conditions induced by stresses imposed by PB plus abiotic stimuli (High Ca, High Fe, Mg deprivation or Low pH); C) The metabolism of glycerol by Klebsiella pneumoniae under the pressure of PB; D) The metabolism of glycerol by Klebsiella pneumoniae under the pressure of PB plus abiotic stimuli (High Ca, High Fe, Mg deprivation or Low pH). (PDF 145 kb) [file 12864_2016_3070_MOESM3_ESM.pdf]

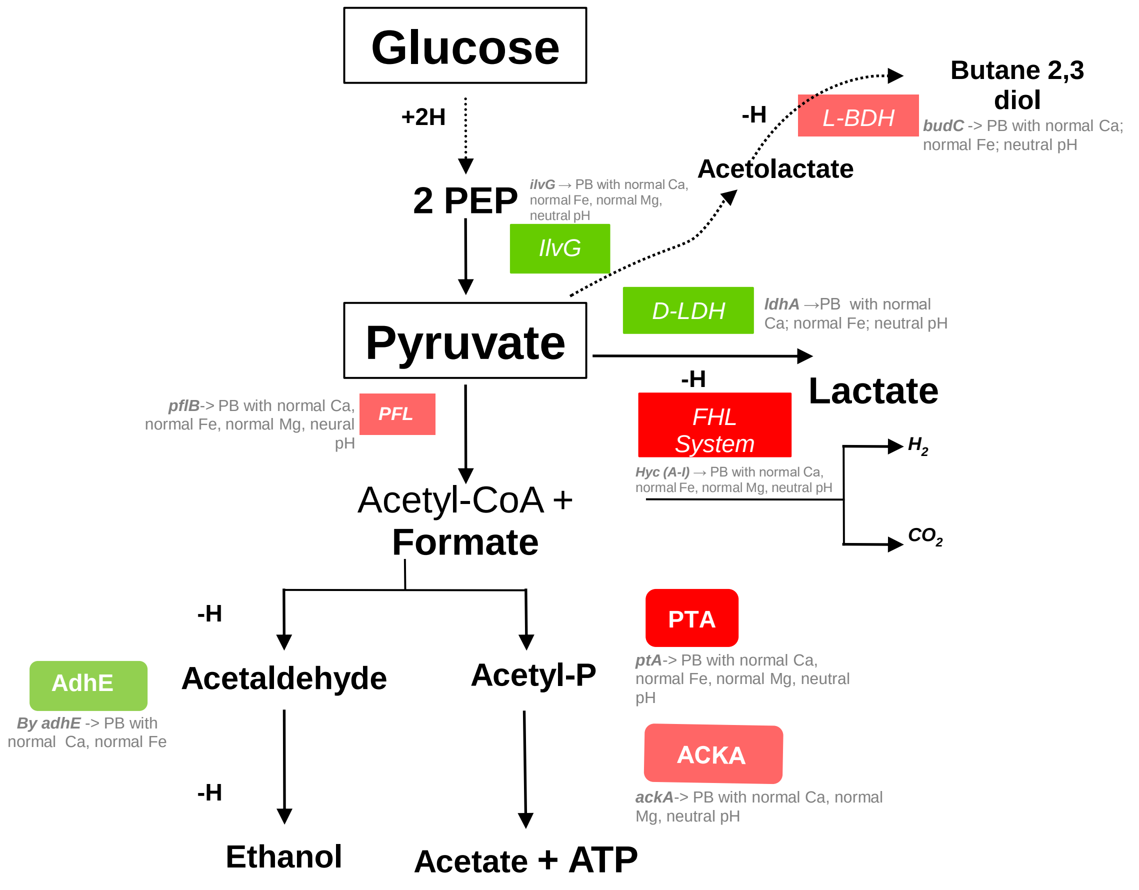

Figure A: Scheme of carbon flux in *Klebsiella pnemoniae* Kp13 under fermentative conditions induced by stresses imposed by PB. Pyruvate can be cleaved by pyruvate formatelyase (PFL), and the citric acid cycle could be interrupted under PB stress and divided into two branches because the expression of genes encoding for 2-oxoglutarate dehydrogenase complex are repressed (down-regulated in PB and PB + abiotic stimuli). +H denotes the generation of reducing equivalents, whereas –H assigns its consumption. ALS, 2-acetolactate synthetase; FHL denotes the activity of the formate hydrogenlyase complex. Up-regulation is depicted as green boxes, whereas down-regulation as red boxes.

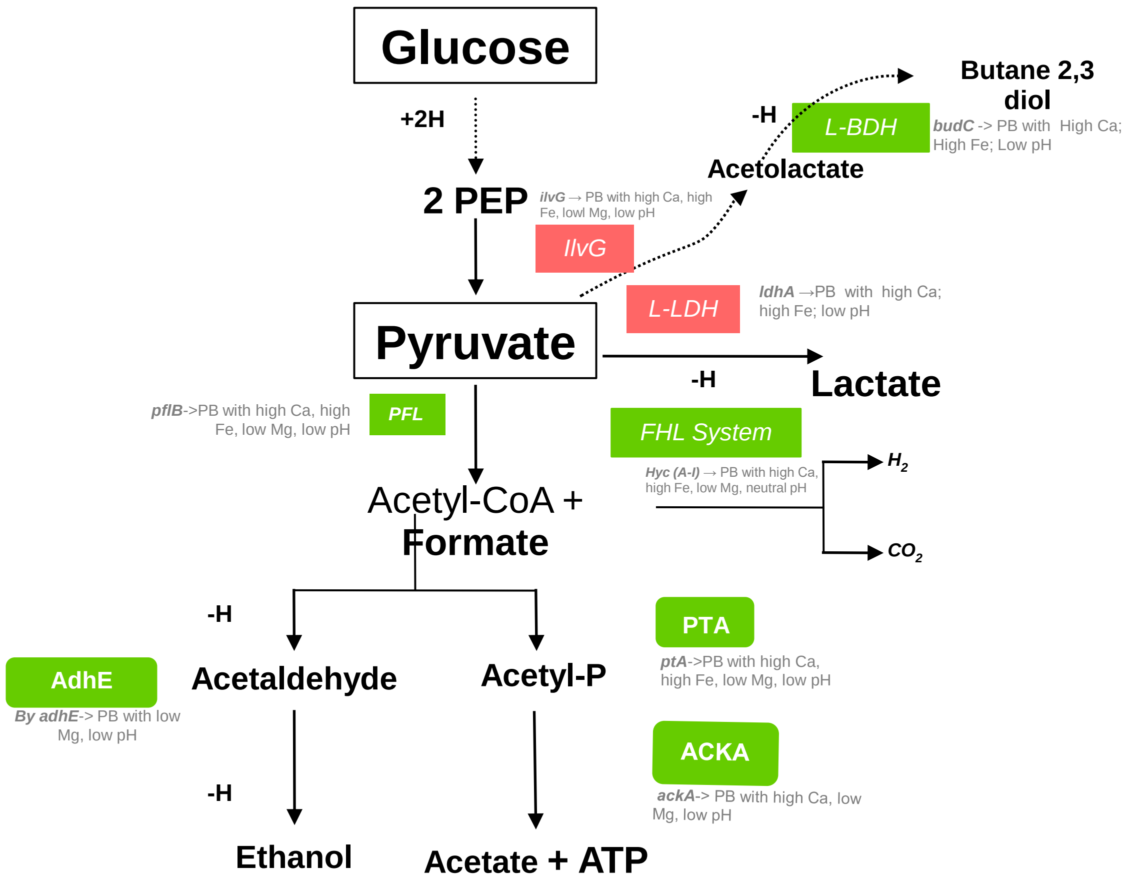

Figure B: Scheme of carbon flux in *Klebsiella pneumoniae* Kp13 under fermentative conditions induced by stresses imposed by PB plus abiotic stimuli (High Ca, High Fe, Mg deprivation or Low pH). Pyruvate can be cleaved by pyruvate formatelyase (PFL), and the citric acid cycle could be interrupted under PB plus abiotic stimuli stresses and divided into two branches because the expression of genes encoding for 2-oxoglutarate dehydrogenase complex are repressed (down-regulated in PB and PB + abiotic stimuli). +H denotes the generation of reducing equivalents, whereas –H assigns its consumption. ALS, 2-acetolactate synthetase; FHL denotes the activity of the formate hydrogenlyase complex. Up-regulation is depicted as green boxes, whereas down-regulation as red boxes.

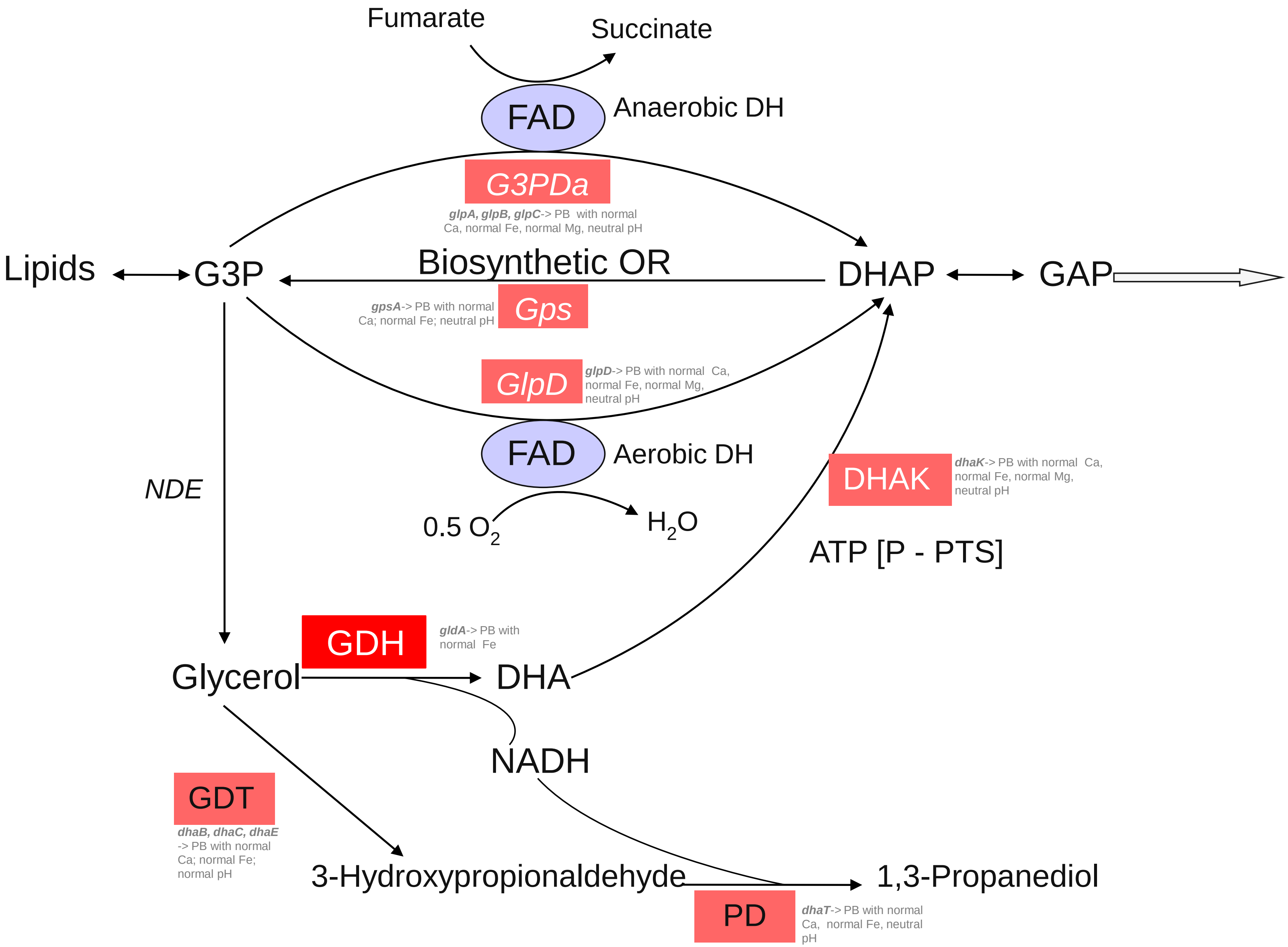

Figure C The metabolism of glycerol by *Klebsiella pneumoniae* under the pressure of PB. Genes encoding for an aerobically induced G3P dehydrogenase (DH) and an anaerobically are down-regulated, and both enzymes are membrane-bound flavoproteins that feed the electrons directly into the respective aerobic or anaerobic respiratory chains. A third enzyme, an oxidoreductase (OR), is responsible for de novo G3P biosynthesis when G3P is not provided in the medium. The lower part of the figure presents the oxidative and reductive branches of glycerol fermentation by *Klebsiella*. The homodimeric dihydroxyacetone kinase (DHAK) from *Klebsiella* uses ATP as substrate, whereas the heterotrimeric enzyme from *E. coli* uses the phosphate from PEP, which is transferred via a cascade of relay proteins to the ADP firmly bound to the ultimate kinase subunit. E. coli possesses the genetic capacity to synthesize the constituent polypeptides of this pathway except that coding for a functional glycerol dehydratase. Transformation with the gene for glycerol dehydratase enables *E. coli* to also ferment glycerol. DHAK, dihydroxyacetone kinase; GD, glycerol dehydrogenase; GDT, glycerol dehydratase; PD, propanediol dehydrogenase, P -PTS indicates that the phosphate for dihydroxyacetone phosphorylation in *E. coli* is transferred from PEP via the phosphotransferase system to dihydroxyacetone kinase.

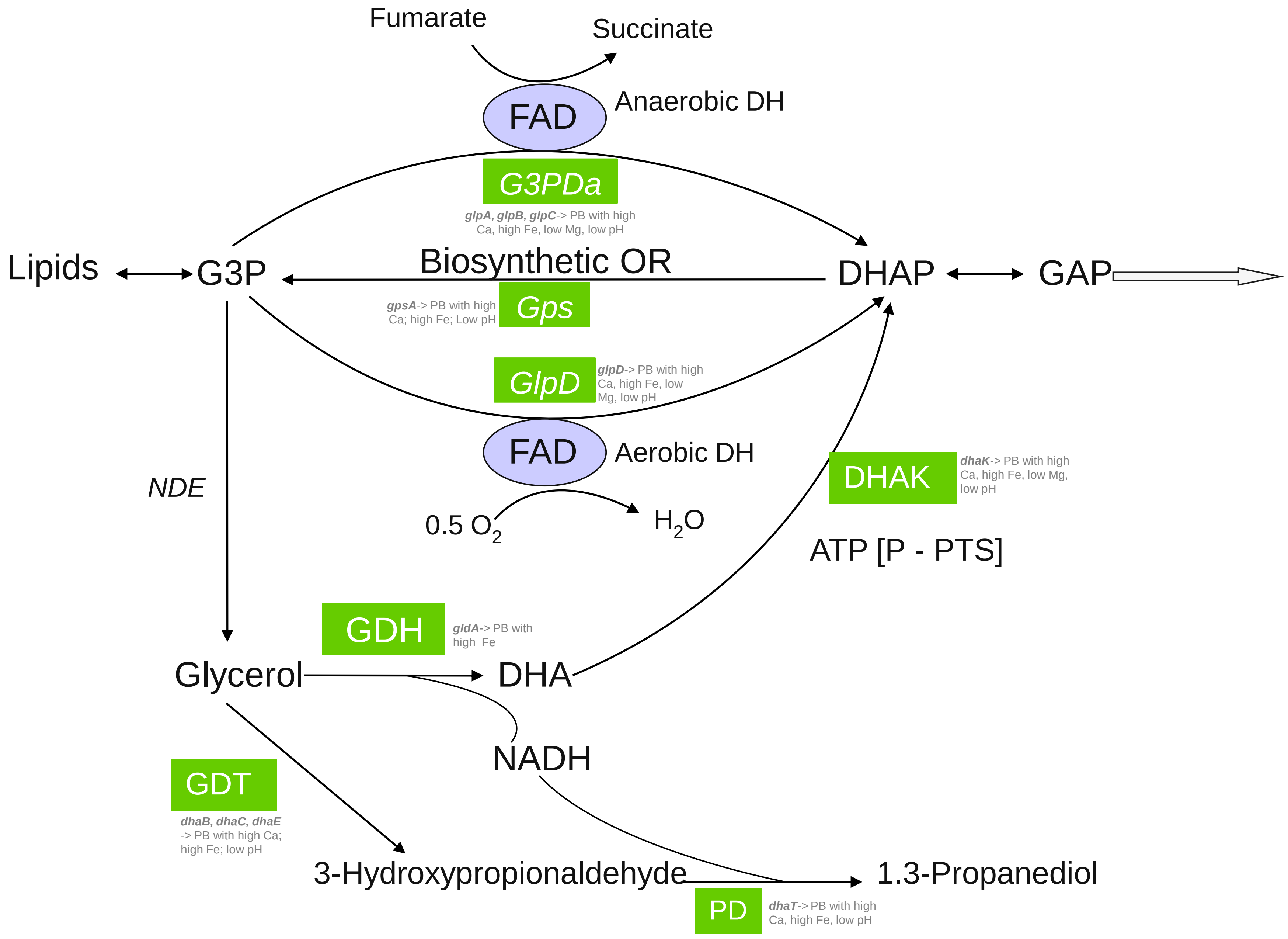

Figure D The metabolism of glycerol by *Klebsiella pneumoniae* under the pressure of PB plus abiotic stimuli (High Ca, High Fe, Mg deprivation or Low pH). An aerobically induced G3P dehydrogenase (DH) and an anaerobically are down-regulated, and both enzymes are membrane-bound flavoproteins that feed the electrons directly into the respective aerobic or anaerobic respiratory chains (PB inhibit the first enzyme complex of respiratory chain). A third enzyme, an oxidoreductase (OR), is responsible for de novo G3P biosynthesis when G3P is not provided in the medium. The lower part of the figure presents the oxidative and reductive branches of glycerol fermentation by *Klebsiella*. The homodimeric dihydroxyacetone kinase (DHAK) from Klebsiella uses ATP as substrate, whereas the heterotrimeric enzyme from *E. coli* uses the phosphate from PEP, which is transferred via a cascade of relay proteins to the ADP firmly bound to the ultimate kinase subunit. *E. coli* possesses the genetic capacity to synthesize the constituent polypeptides of this pathway except that coding for a functional glycerol dehydratase. Transformation with the gene for glycerol dehydratase enables E. coli to also ferment glycerol. DHAK, dihydroxyacetone kinase; GD, glycerol dehydrogenase; GDT, glycerol dehydratase; PD, propanediol dehydrogenase, P -PTS indicates that the phosphate for dihydroxyacetone phosphorylation in E. coli is transferred from PEP via the phosphotransferase system to dihydroxyacetone kinase.
