# Supplementary figures and images for: The polymyxin B-induced transcriptomic response of a clinical, multidrug-resistant Klebsiella pneumoniae involves multiple regulatory elements and intracellular targets
Source: BMC Genomics. 2016 Oct 25;17(Suppl 8):737. doi: 10.1186/s12864-016-3070-y (PMC5088521; doi:10.1186/s12864-016-3070-y)

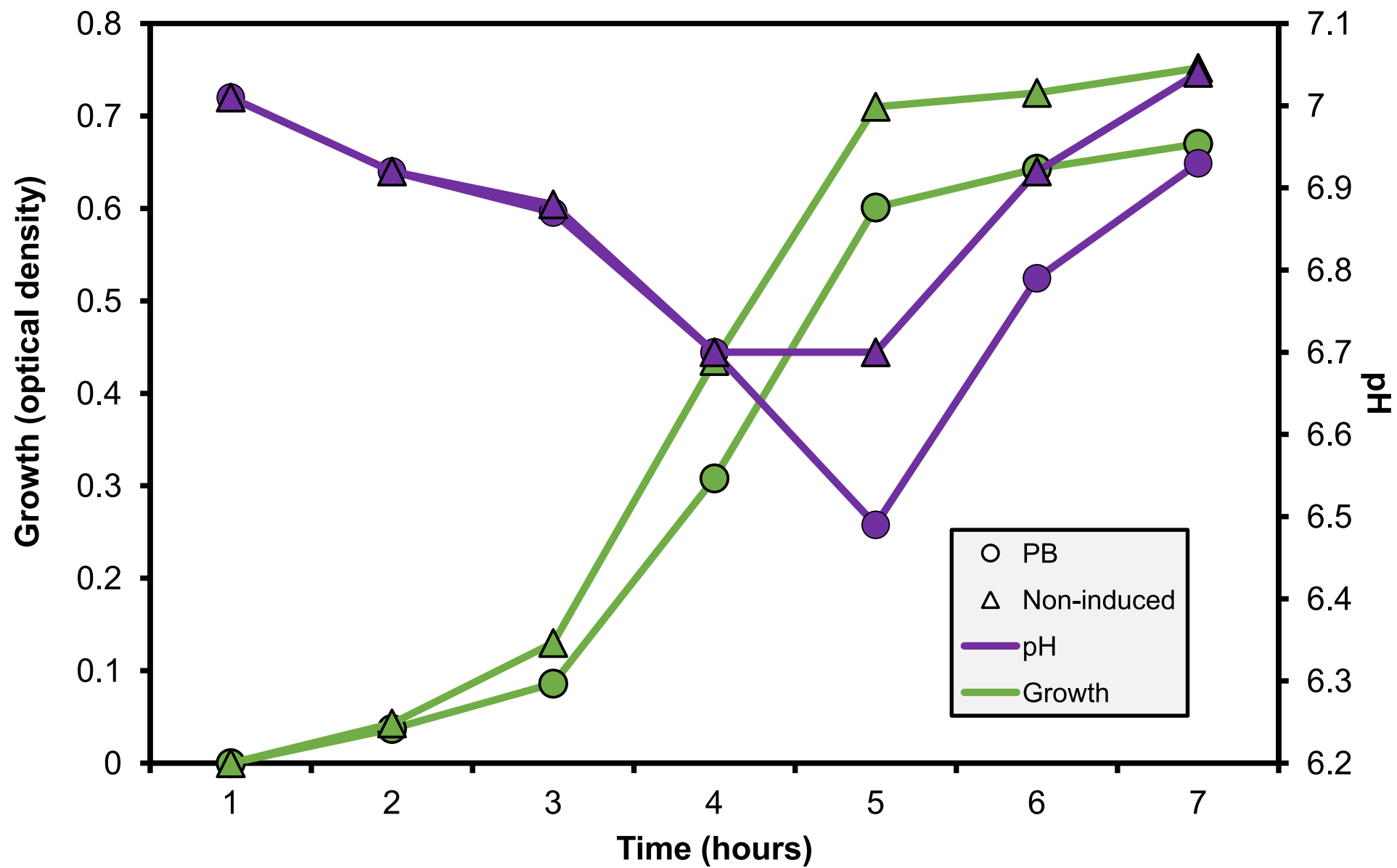

Supplement: Additional file 4: — Klebsiella pneumoniae Kp13 growth under polymyxin B (PB) exposure vs. non-induced bacteria grown without PB. Description: Bacterial growth and medium pH were recorded at hourly intervals at seven timepoints. (PDF 46 kb) [file 12864_2016_3070_MOESM4_ESM.pdf]

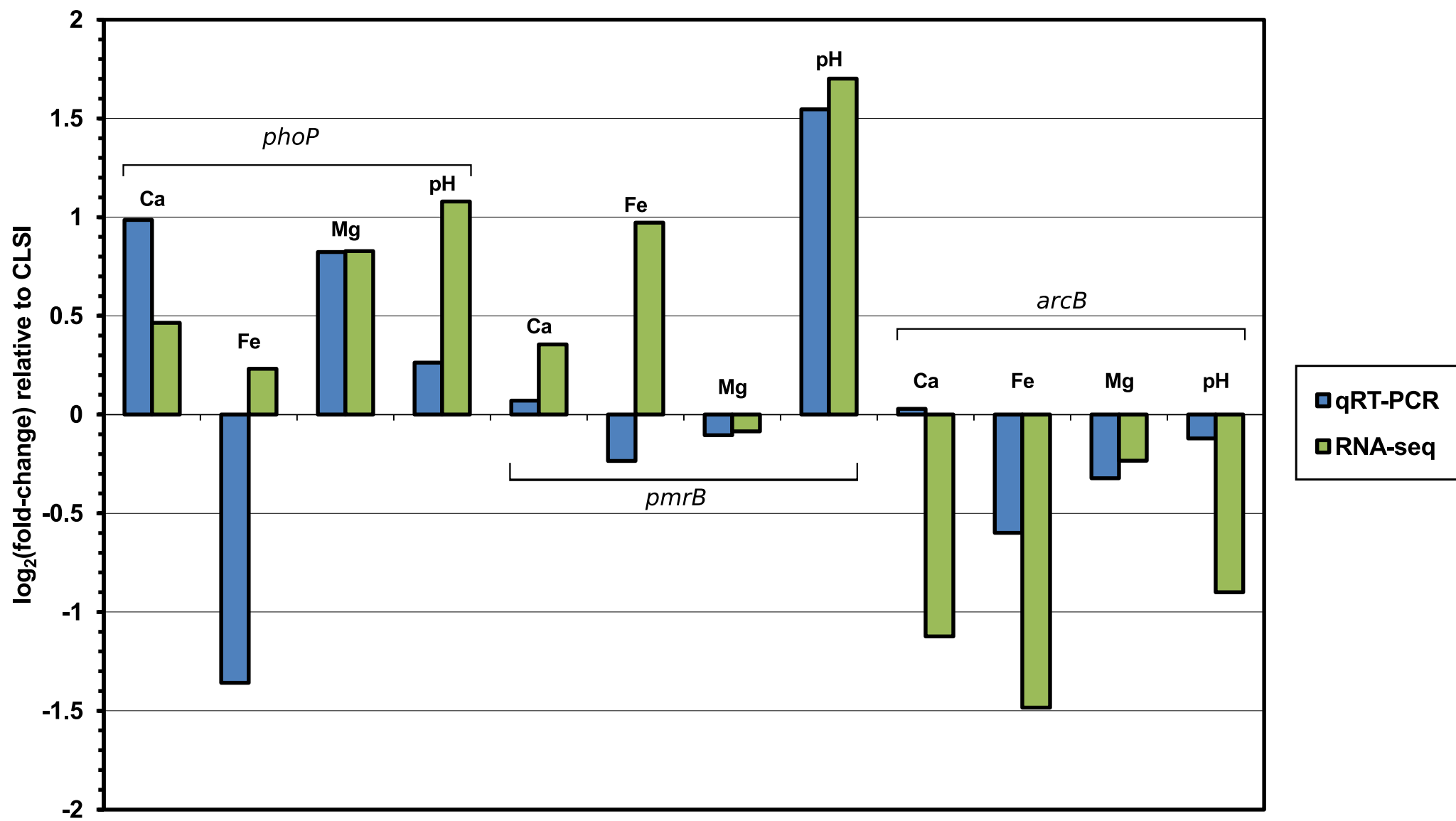

Supplement: Additional file 5: — Validation of the expression of pmrB, arcA and phoB using qRT-PCR. Description: The log2-transformed expression values of each gene, relative to the PB condition (strain Kp13PolB), is reported for both the RNA-seq data and qRT-PCR measurements. (PDF 40 kb) [file 12864_2016_3070_MOESM5_ESM.pdf]

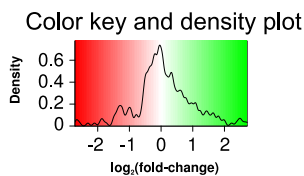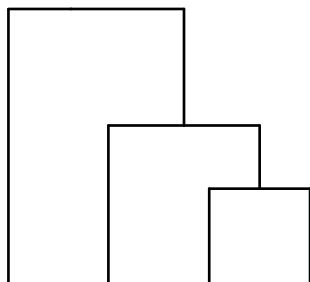

**Genes**

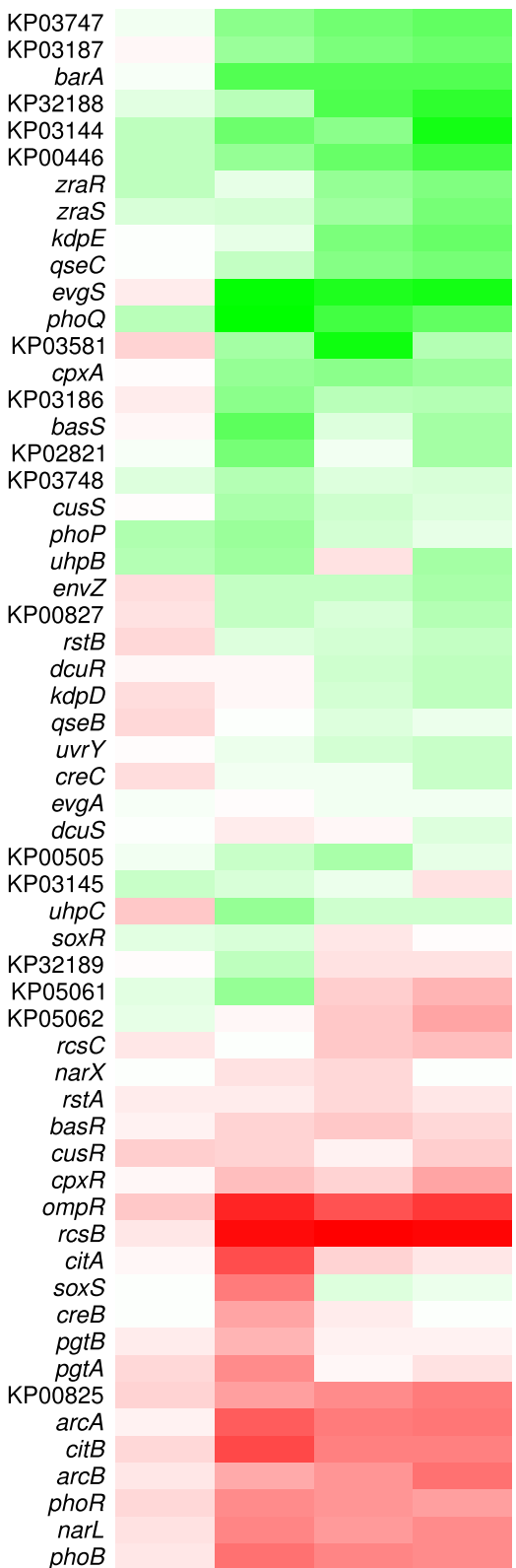

No Mg

Low pH

High Ca

High Fe

**Conditions**

Supplement: Additional file 6: — Expression heatmap of regulatory systems. Description: Expression values are represented as log2(fold-change) of the condition compared to the PB condition (log2(abiotic condition/PB condition). Thus, a positive log2FC value indicates higher expression of the gene in face of abiotic stress to which it was subjected. In this figure, all genes that we identified as a possible two-component regulatory system (by sequence similarity, conserved domains, genomic positional evidence and co-occurrence) are depicted. Genes for which we failed to identify a known homolog in the literature and databases are only cited by their locus tag in Kp13 strain. (PDF 79 kb) [file 12864_2016_3070_MOESM6_ESM.pdf]
